# Comparative transcriptome analyses define genes and gene modules differing between two *Populus* genotypes with contrasting stem growth rates

**DOI:** 10.1186/s13068-020-01758-0

**Published:** 2020-08-09

**Authors:** Xiao Han, Yi An, Yangyan Zhou, Chao Liu, Weilun Yin, Xinli Xia

**Affiliations:** 1 State Key Laboratory of Subtropical Silviculture, College of Forestry and Biotechnology, Zhejiang A&F University, Lin’an, Hangzhou, 311300 China; 2 Sino-Australia Plant Cell Wall Research Centre, State Key Laboratory of Subtropical Silviculture, College of Forestry and Biotechnology, Zhejiang A&F University, Lin’an, Hangzhou, 311300 China; 3 Beijing Advanced Innovation Center for Tree Breeding by Molecular Design, National Engineering Laboratory for Tree Breeding, College of Biological Sciences and Technology, Beijing Forestry University, Beijing, 100083 China

**Keywords:** *Populus*, Stem radial growth, Cell cycle, Cell division, Secondary vascular tissue, Co-expression network, CRISPR/Cas9

## Abstract

**Background:**

Wood provides an important biomass resource for biofuel production around the world. The radial growth of tree stems is central to biomass production for forestry and biofuels, but it is challenging to dissect genetically because it is a complex trait influenced by many genes. In this study, we adopted methods of physiology, transcriptomics and genetics to investigate the regulatory mechanisms of tree radial growth and wood development.

**Results:**

Physiological comparison showed that two *Populus* genotypes presented different rates of radial growth of stems and accumulation of woody biomass. A comparative transcriptional network approach was used to define and characterize functional differences between two *Populus* genotypes. Analyses of transcript profiles from wood-forming tissue of the two genotypes showed that 1542, 2295 and 2110 genes were differentially expressed in the pre-growth, fast-growth and post-growth stages, respectively. The co-expression analyses identified modules of co-expressed genes that displayed distinct expression profiles. Modules were further characterized by correlating transcript levels with genotypes and physiological traits. The results showed enrichment of genes that participated in cell cycle and division, whose expression change was consistent with the variation of radial growth rates. Genes related to secondary vascular development were up-regulated in the faster-growing genotype in the pre-growth stage. We characterized a BEL1-like (BELL) transcription factor, *PeuBELL15*, which was up-regulated in the faster-growing genotype. Analyses of transgenic *Populus* overexpressing as well as CRISPR/Cas9-induced mutants for *BELL15* showed that *PeuBELL15* improved accumulation of glucan and lignin, and it promoted secondary vascular growth by regulating the expression of genes relevant for cellulose synthases and lignin biosynthesis.

**Conclusions:**

This study illustrated that active division and expansion of vascular cambium cells and secondary cell wall deposition of xylem cells contribute to stem radial increment and biomass accumulation, and it identified relevant genes for these complex growth traits, including a BELL transcription factor gene *PeuBELL15*. This provides genetic resources for improving and breeding elite genotypes with fast growth and high wood biomass.

## Background

*Populus* is a model system for forest tree growth and development. It has a fully sequenced genome that enables new approaches for the dissection of complex, quantitative growth and yield traits important for industrial forestry and biofuel production. The understanding of plant growth dynamics is becoming increasingly important. The perennial *Populus* has distinct active and dormant periods that influence overall stem growth rates and wood yield. At the beginning of the growing season, named the ‘pre-growth’ phase, trees end dormancy and exhibit the onset of growth. Trees then enter the ‘fast-growth’ phase, which has a high growth rate. In response to environmental cues including water availability and day length, growth rates gradually decline and trees go into the ‘post-growth’ phase. Eventually, they re-enter dormancy. Consequently, the variation of growth parameters of trees presents an S-type trend (slow–fast–slow) during the active period. Previous research has explored the relationships between the physiological characteristics of *Populus* and different growth rates or growing conditions. They also have showed that *Populus* plants display an S-type growth model [1–3], but the molecular genetic basis of the regulation of growth rates is poorly understood. Therefore, it would be of great importance to connect the physiological changes of *Populus* growth with the molecular data.

Woody xylem is the main reservoir of tree biomass, and secondary growth in the stem confers woody biomass accumulation [4, 5]. Secondary growth is the process of radial growth of tree stems. The stem lateral meristem, the vascular cambium, gives rise to both secondary xylem (wood) and secondary phloem [5]. Cell division in the cambium is a primary determinant of the rate of stem radial growth, and it is influenced by environmental conditions, as can be seen in tree rings [6–8]. However, the mechanisms that regulate cambium cell division and ultimately stem growth and wood yield are complex, and they are presumably influenced by actions of large numbers of genes.

Advances in genomics and computational biology offer the ability to model the coordinated actions of large amounts of genes underlying complex traits such as stem growth rates. For tree biology, the completion of the *Populus trichocarpa* genome [9] and related data releases [10] provide the resources required to identify and unravel transcriptional regulation associated with *Populus* stem growth and development. Studies using microarray experiments have investigated the transition between cambial activity and dormancy utilizing cryosection-isolated cambial cells from the woody plant *Populus tremula* [6, 7]. Their results showed that the different stages of secondary xylem development were strongly correlated with changes in gene expression. The transition from primary to secondary growth in the poplar stem also has been analyzed by genomic microarray. That work identified the different expression patterns of regulatory factors and genes related to cell wall biogenesis during stem development, and it identified the dominant genes in the primary and secondary growth region [11]. Poplar transcript arrays for stem growth variation were constructed under water deficit [12] and elevated CO_2_ concentration [13]. The results showed that water and CO_2_ conditions significantly changed the transcriptomic profiles of stems, and that genes related to cell wall metabolisms, hormone responses and transcriptional regulation were correlated with the physiological variation of plants. Recently, the availability of advanced next-generation high-throughput sequencing has accelerated and improved tissue-specific gene profiling and comparative genomic or transcriptomic analysis in plant growth [14, 15]. These sequencing technologies necessitate and enable new computational approaches for extracting information about how genes interact to influence complex traits [16, 17]. One powerful approach is gene co-expression analysis, which identifies ‘modules’ of genes that show similar expression patterns across diverse conditions and tissues or in response to experimental treatment [18]. Because genes that show a strong co-expression relationship often participate in related biological processes, this approach allows the assignment of genes to putative functional groups without prior knowledge. Gene modules can be further characterized through correlations with traits of interest and experimental variables or by determining the enrichment of genes of specific biological functions within modules [18].

*Populus* clones with different genotypes display varied growth rates [3], demonstrating the genetic regulation of this trait. A fast-growing hybrid *Populus* clone Neva (*Populus* × *euramericana* cv. ‘Neva’) was introduced and cultivated in China in the 1980s. Its characteristics of fast growth and short rotation cycle make it one of the most important plantation clones in the north of China. A control clone, I-214 (*Populus* × *euramericana* cv. ‘I-214’), was introduced into China in the 1960s. Compared with I-214, Neva exhibits more rapid height and stem radial growth, resulting in greater woody biomass. In this study, we compared and contrasted the transcriptional differences during stem growth in two *Populus* genotypes Neva and I-214 with contrasting stem growth rates. Cambial transcript levels were determined for Neva and I-214 at three growth phases. Stage-specific sampling, together with detailed physiological and growth analyses, provided candidate biological processes and expressed genes that supported cambial activity and stem growth. Co-expression network analysis identified some specific gene modules correlated with differences in genotypes and growth traits. In addition, a BEL1-like transcription factor gene was identified through detailed dissection of a gene module showing strong genotypic correlations and was characterized using transgenics as affecting secondary vascular growth.

## Results

### Faster stem radial growth and higher biomass accumulation in Neva than in I-214

**Fig. 1:**
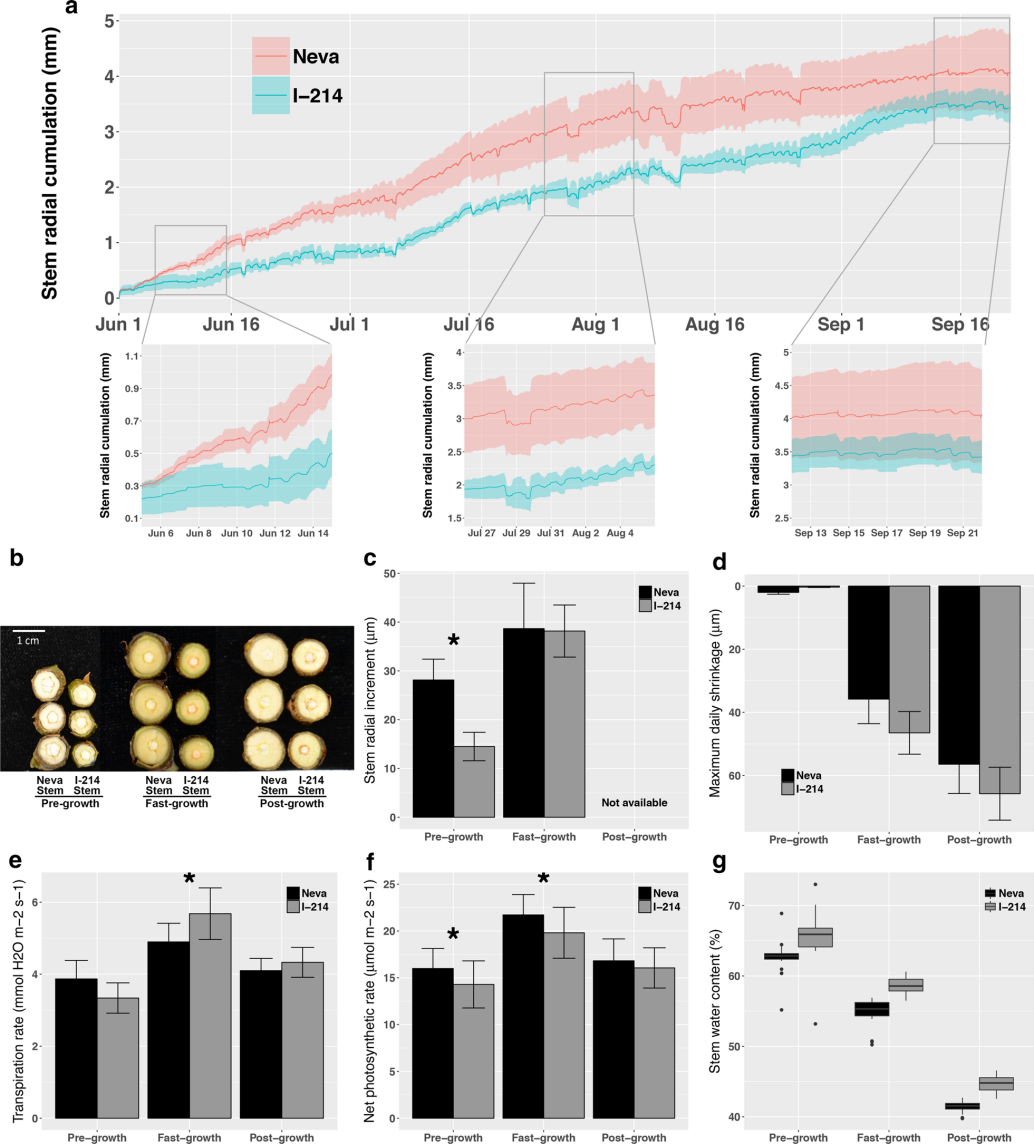
Physiological comparison between the faster-growing Neva and control I-214. **a** The change in stem radius of the two genotypes from June to September, as measured using a point dendrometer. The three expanded plots provide higher resolution for variation in different growth stages. **b** Representative stems from the two genotypes in the pre-growth, fast-growth and post-growth stages. **c** The stem radial daily increment of the two genotypes in three growth stages. **d** The maximum daily shrinkage of the two genotypes in three growth stages. **e** The transpiration rates of the two genotypes in three growth stages. **f** The maximum net photosynthetic rates of the two genotypes in three growth stages. **g** The stem relative water contents of the two genotypes in three growth stages

To compare the differences in growth between Neva and I-214, real-time diameter dendrometers were used for continuous measurement of the stem radial variation of the two genotypes over one growing season. Despite issues of shrinking and swelling, dendrometer data taken at minutes intervals accurately documented both the timing and rate of growth [19]. The diameters of stems fluctuated daily, and they steadily increased from June to September (Fig. 1a). The two genotypes displayed distinct growth patterns. At the beginning of June (the pre-growth phase), I-214 exhibited only slight stem variation, while Neva presented significant stem increment without pronounced daily contraction (Fig. 1b–d). Both genotypes had high stem growth rates at the beginning of August (the fast-growth phase), and their stems displayed a typical rhythm of diurnal contraction and expansion. I-214 showed a higher maximum daily shrinkage (MDS) than Neva, consistent with the higher transpiration rates of I-214 (Fig. 1e). By the end of September (the post-growth phase), the growth of both genotypes produced plateaus and was relatively stable. During the growth season, Neva presented higher increments of stem radial growth and net photosynthetic rates than I-214 (Fig. 1f). The stem water content (SWC) of the two genotypes decreased with stem growth. Neva showed lower SWC than I-214, indicating a higher accumulation of dry biomass (Fig. 1g). The sections of different internodes showed more xylem cells in Neva than in I-214 at the 4th, 7th and 10th internodes from the apex, respectively (Additional file 1: Figure S1A). The wood chemical composition analysis showed that Neva had higher contents of lignin and glucan in the stem than I-214 (Additional file 1: Figure S1B, S1C).

### Transcriptomic variation of *Populus* cambium between growth stages and genotypes

To interpret the transcriptomic variation between two *Populus* genotypes with different growth rates throughout the annual growth period, 18 gene expression profiles were constructed from the two genotypes with three replicates each at three growth stages. After trimming adapter sequences and removing low-quality reads and multi-matched reads, the Illumina platform generated 2.2–8.7 million unique clean reads from each of these libraries. The clean reads were all mapped to the *Populus trichocarpa* referenced genome. Between 1.7 and 5.3 million unique matched reads were used for further detailed investigation of gene expression in various samples at different growth stages. In total, 34,937 mapped genes were identified in all growth stages combined with Neva (33,622) and I-214 (33,624) (Additional file 2: Data S1) accounting for 84.52% of the available 41,335 genes in the *Populus* genome [9].

**Fig. 2:**
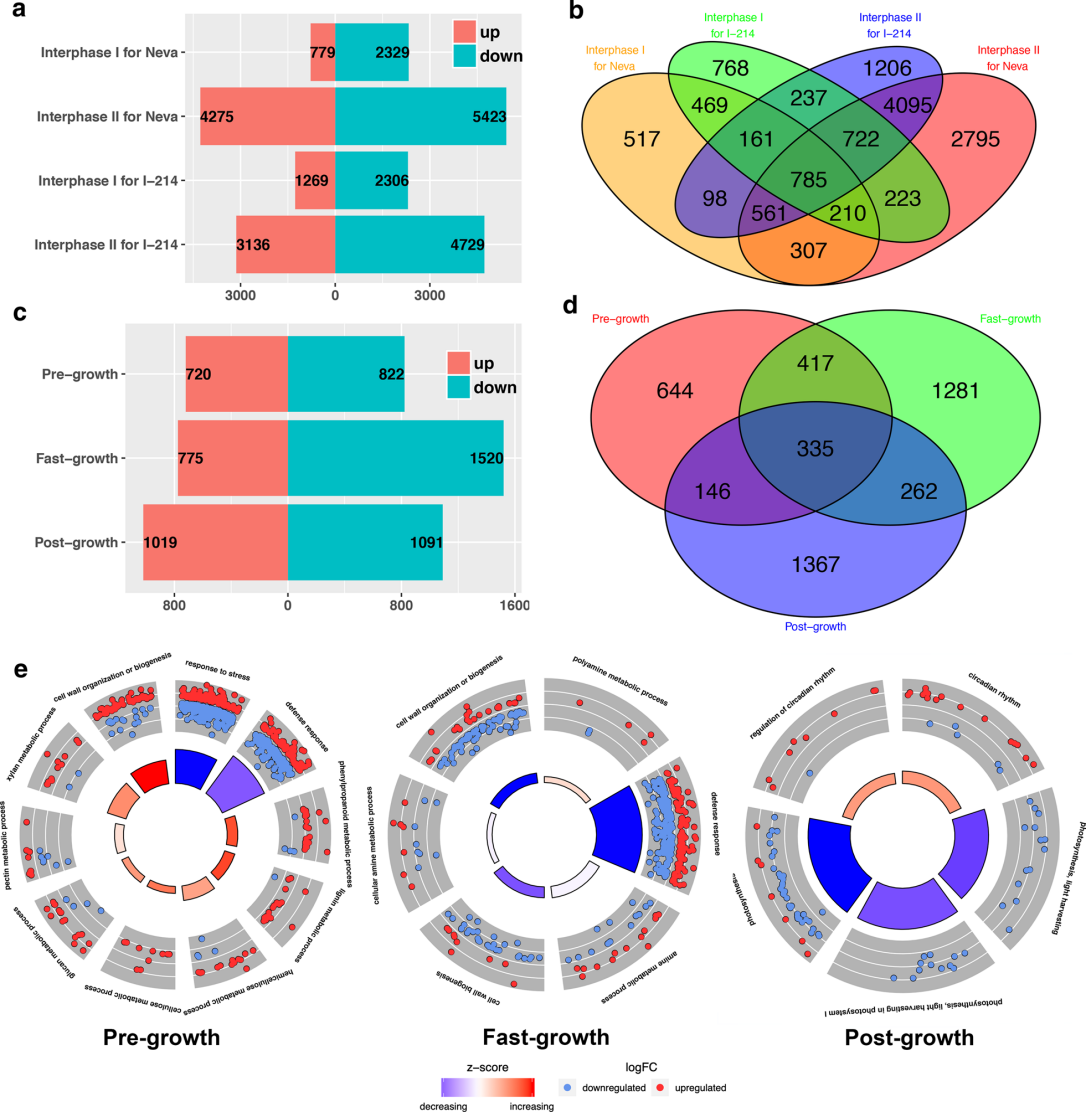
Differentially expressed genes between different growth stages and the two genotypes Neva and I-214. **a** The differentially expressed genes in interphase I (the transition from the pre-growth to the fast-growth stage) and interphase II (the transition from the fast-growth to the post-growth stage) for the two genotypes. **b** The Venn diagram presents genes representation in interphase I and interphase II for the two genotypes. **c** The differentially expressed genes between the two genotypes in three growth stages. **d** The Venn diagram presents genes representation between the two genotypes in three growth stages. **e** The gene ontology annotation of differentially expressed genes enriched in the pre-growth, fast-growth and post-growth stages. The outer circle presents a scatter plot for each GO term of the logFC of differentially expressed genes. Red points mean up-regulated genes and blue ones down-regulated genes. The inner circle shows a z-score, which represent a hint if certain GO term is more likely to be decreased (blue) or increased (red)

To understand changes in gene expression during the transitions in growth periods for each genotype, we identified differentially expressed genes (DEGs) between one time point and the preceding time point. These genes signified a transition in gene expression in interphase I (pre-growth stage to fast-growth stage) and interphase II (fast-growth stage to post-growth stage) (Fig. 2a, b; Additional file 3: Figure S2; Additional file 4: Data S2). To further elucidate the regulatory variation of biological processes during the growth period, we classified these genes into functional categories according to gene ontology (GO) (Additional file 5: Data S3). For Neva in interphase I, most of the 779 up-regulated DEGs were enriched in cellular macromolecule metabolic process (GO:0044260), nucleobase-containing compound metabolic process (GO:0006139) and cellular component organization or biogenesis (GO:0071840). Most of the 2329 down-regulated DEGs were involved in metabolic processes (GO:0008152) and response to stimuli (GO:0050896). In interphase II, metabolic processes and response to stimuli had most of the 4275 up-regulated genes, and biological regulation and cellular component organization or biogenesis enriched many down-regulated genes. For I-214, in interphase I, up-regulated DEGs were mainly enriched in macromolecule metabolic processes (GO:0043170) and cellular component organization or biogenesis, while down-regulated genes were involved in response to stimuli and biological regulation. During interphase II, many regulated DEGs were clustered in metabolic processes and response to stimuli, and down-regulated genes were enriched in biological regulation and cellular component organization or biogenesis, the same as for Neva (Additional file 6: Figure S3).

In addition, the analyses of genotype-dependent DEGs showed that there were 1542, 2295 and 2110 DEGs between the two genotypes in the three growth phases, respectively (Fig. 2c, d; Additional file 7: Data S4). Comparison of gene expression levels in the growth stages revealed a massive reprogramming of gene expression in the fast-growth phase, with more DEGs upregulated in I-214 (Additional file 3: Figure S2). GO enrichment analysis also was used for functional interpretation (Additional file 8: Data S5). In the pre-growth stage, the faster-growing Neva expressed abundant genes involved in cell wall-related biogenesis (GO:0009832, GO:0009834, GO:0042546, GO:0071554, and GO:0071669), especially in the biosynthetic processes of lignin (GO:0009808 and GO:0009809) and cellulose (GO:0030243). By comparison, more genes were predominantly clustered in the stress and defense response processes (GO: 0006950, GO:0006952, and GO:0050896) in I-214. When moving forward towards the fast-growth stage, several receptor protein signaling pathways (GO:0007167 and GO:0007169) occurred in Neva, as were polyamine biosynthetic processes (GO:0006595 and GO:0006596). For I-214, except remaining stimuli (GO:0050896) and defense response, several cell wall biogenesis processes (GO:0009834 and GO:0042546) emerged. Until the post-growth stage, Neva enriched more genes in the circadian rhythm processes (GO:0007623, GO:0042752 and GO:0048511). In I-214, some functions involving the response to light (GO:0009765, GO:0009768, and GO:0015979), as well as stress and stimuli response, had more genes (Fig. 2e). Based on the annotation of total 2581 *Populus* transcription factors [20], 99, 153 and 172 differentially expressed transcription factors were identified in the pre-growth, fast-growth and post-growth stages, respectively (Additional file 9: Data S6). The MYB, bHLH, Homeobox, NAC, AP2/ERF, C2H2, and MADS genes constituted a larger proportion. Every gene family of transcription factors had variable expression patterns in the different growth phases. However, some exceptions like Homeobox (Potri.009G009900), MYB (Potri.003G144300), MADS (Potri.003G170000), NAC (Potri.014G075900) and C2H2 (Potri.014G134400) family members had the same up-regulated pattern in the faster-growing genotype during all three growth phases.

### Gene modules related to cell growth and cell wall biosynthesis revealed by co-expression network

**Fig. 3:**
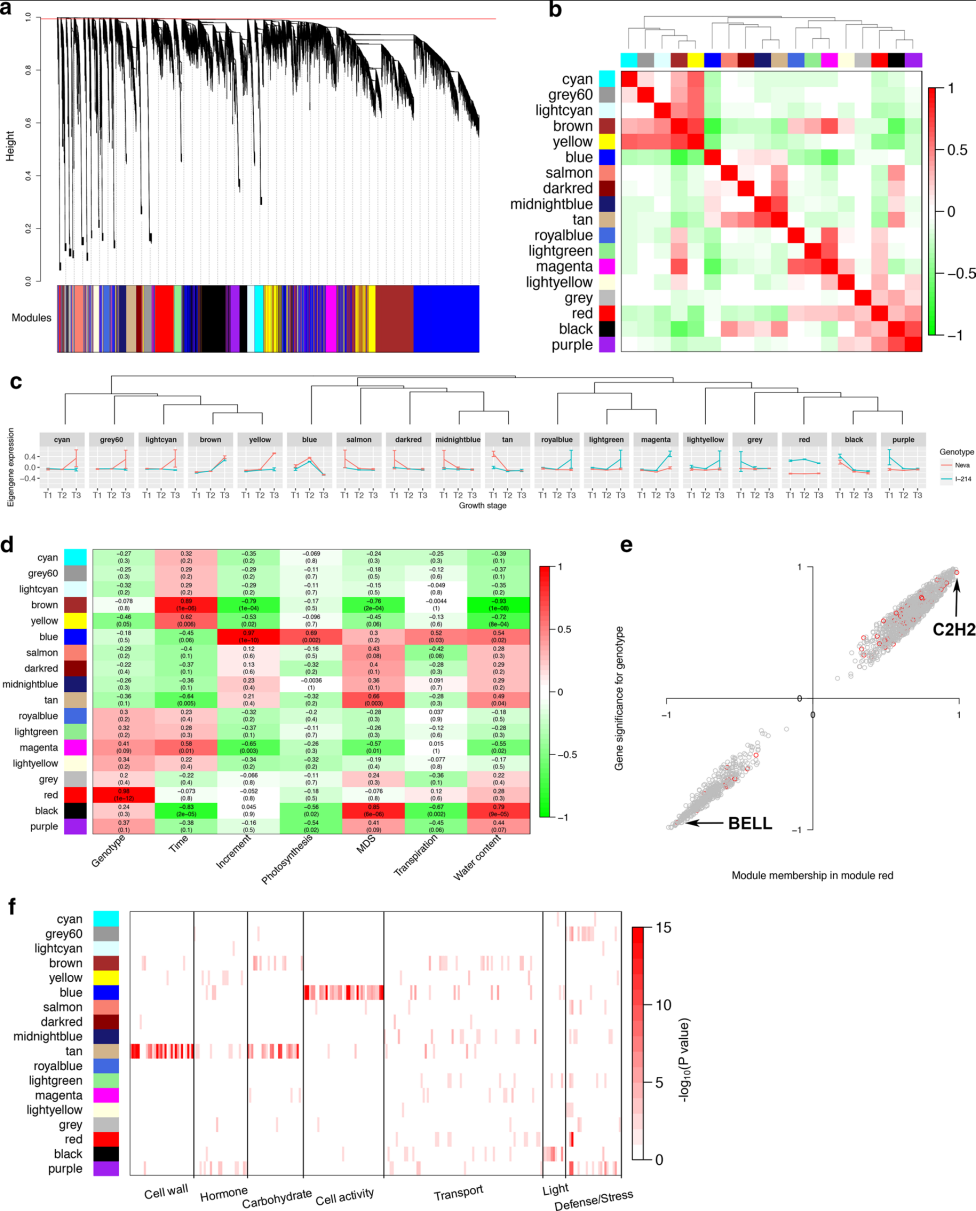
Gene co-expression network analysis for genes expressed in stems across growth timepoints for genotypes Neva and I-214. **a** Clustering dendrogram of all expressed genes together with assigned module colors. **b** The correlation between modules. Each row and column correspond to a module eigengene. The table is color-coded by the correlation according to the color legend. **c** The expression patterns of eigengene in modules. **d** The module–trait associations. Each row corresponds to a module eigengene, column to a trait. Each cell contains the corresponding correlation and *p*-value. The table is color-coded by correlation according to the color legend. **e** A scatterplot of gene significance (GS) for genotype vs module membership (MM) in the module red. There is a highly significant correlation (Cor = 1, *p* < 1e-200) between GS and MM in this module, illustrating that genes highly significantly associated with genotype trait also are the most important elements of modules associated with the trait. The red points represent transcription factors, and the grey ones are other genes in the module red. **f** The heatmap for the enrichment of genes in modules for GO terms from the cell wall, hormone, carbohydrate metabolism, cell activity, transport, light, and the response of defense and stress

To further represent a global view of transcriptional patterns through the growth period, a weighted correlation network analysis was used to define clusters (modules) of co-regulated genes that varied in the two clones throughout the growing season. Modules were originally characterized by gene interconnection and recalculation by merging adjacent modules whose expression profiles were similar (Fig. 3a). A total of 18 modules were finally detected to have a high similarity of expression with a minimum module size of 400. They were assigned individual colors and categorized into four clusters by hierarchical cluster analysis and correlations across all combinations of modules (Fig. 3b).

To elucidate the variation of gene expression in the two genotypes during the growth stages, we constructed the expression profiles for every module (Fig. 3c). As visualized by eigengene values for each module, variation in transcript levels across growth stages was the primary factor in defining gene co-expression modules, followed by the genotypes. Eighteen modules displayed diverse expression profiles for the two genotypes at different growth stages. In cluster I, comprising modules cyan, grey60, lightcyan and yellow, the expression of clustered genes increased in Neva in the post-growth stage. For cluster II, comprising modules salmon, dark red, midnight blue and tan, higher expression values of eigengenes of Neva presented in the pre-growth phase. To identify modules that were the most closely associated with the measured physiological traits, we correlated the eigengenes that summarized module profiles with external traits to look for the most significant associations. Correlations were computed between each gene module and phenotypic trait. Interestingly, significant correlations were found between each trait and at least one module (Fig. 3d). For example, the module red, with a high correlation with genotype trait, showed significantly different gene expression in the two genotypes throughout the growth period. Genes in this module were scatter-plotted based on the significance of their correlation with genotype and module membership (the value of gene connectivity in one module), and transcription factors in module red were marked on the plot (Fig. 3e). The transcription factor C2H2 zinc finger protein (Potri.014G134400) with the highest positive correlation was up-regulated in slow-growth I-214 in the three stages. Conversely, the transcription factor Homeobox BELL gene (Potri.009G009900) with the highest negative correlation expressed higher throughout the growth period in fast-growth Neva. Its ortholog in *Arabidopsis, POUND*-*FOOLISH* (*PNF*), is well known to regulate meristem activity [21, 22].

To facilitate a biological interpretation, modules were identified with significant functional enrichment for specific GO categories (Fig. 3f; Additional file 10: Data S7), to further focus on the functional features of modules. Some important processes related to the development and growth of stems were clustered in distinct modules. The ontologies of genes in module blue with the most expressed genes (9714) embodied many processes related to cell cycle and division, such as DNA metabolic processes (GO:0006259), chromosome organization (GO:0051276), nuclear division (GO:0000280), organelle organization (GO:0006996), and cytoskeleton organization (GO:0007010) (Additional file 11: Figure S4). The eigengene expression level of module blue increased with the amount of growth time, peaked in the fast-growth stage, and then decreased in the post-growth phase. This showed a paralleled variation trend of expressions in the two genotypes (Fig. 3c). The module eigengenes of Neva presented higher expression than I-214. The hub genes were selected based on module membership and the proximity of the expression profiles to that of the corresponding module eigengene. In the pre-growth stage, processes of DNA structure modification and replication clustered more up-regulated DEGs. The MiniChromosome Maintenance protein gene (*MCM*; Potri.001G07400) and Replication Protein A gene (*RPA*; Potri.015G057300), the two hub genes with the high module memberships and up-regulation in Neva, were both implicated in DNA replication [23, 24]. Membrane receptor signaling was improved in the fast-growth stage, which was supported by the presentation of abundant up-regulated leucine-rich repeat protein kinases with high module membership. By the post-growth stage, massive negative regulation of cell activities, like organelle organization and cell cycle-related processes displayed many DEGs. The more up-regulated differentially expressed genes presented in I-214. The results showed that the faster-growing Neva showed improved cell activities during the growth period and retarded the negative regulation of cell activities at the end of the period, to increase the growth rate.

The module tan enriched distinct ontology terms of cell wall biosynthesis and carbohydrate metabolism. A majority of the 253 DEGs out of 1006 genes in module tan presented significantly up-regulated in the pre-growth stage. These DEGs clustered a series of processes associated with cell wall organization and biogenesis, such as metabolism and biosynthesis of lignin, hemicellulose, cellulose and pectin, as well as polysaccharide biosynthesis. Among these DEGs, 25 transcription factor genes were detected, most of which had high degrees of connectivity (Additional file 12: Figure S5A; Table 1). These transcription factors mainly included MYB (seven genes), Homeobox (six genes) and MADS (three genes) family genes, which are reported to participate in plant growth and development [25–27]. Ranking by connectivity, the top 10 hub genes of this module included two transcription factors MYB gene *MYB85* (Potri.015G129100) and Homeobox KNOTTED-like (KNOX) gene *PoptrKNAT* (Potri.001G112200). *Arabidopsis* orthologs of these two genes were *AtMYB85* (At4G22680) and *AtKNAT7* (At1G62990), and both were both involved in secondary cell wall biosynthesis [28, 29]. In the gene co-expression network composed of several top hub genes, a COBRA-like IRREGULAR XYLEM (IRX) protein gene *IRX6* (Potri.015G060100) was co-connected with *MYB85* and *PoptrKNAT* (Additional file 12: Figure S5B). The *IRX* was involved in secondary cell wall biosynthesis with modification of the levels of cellulose and cell wall polysaccharide [30]. Four cellulose synthase (CESA) genes (Potri.006G181900, Potri.018G103900, Potri.004G059600 and Potri.011G069600), two cellulose synthesis-related TRICHOME BIREFRINGENCE-like (TBL) protein genes (Potri.008G069900 and Potri.010G187600), and a lignification-related laccase (LAC) gene (Potri.010G183500) also presented high degrees of connectivity in this co-expressed network. In addition, two calcium-related protein genes *cyclic nucleotide*-*gated channel* (*CNGC*; Potri.017G089900) and *calcium exchanger* (*CAX*; Potri.001G251200), showed a high co-expression pattern with *CESA* and *IRX* genes. These genes were all up-regulated in the faster-growing Neva, and they could contribute to improving secondary cell wall biosynthesis and polysaccharide deposition.

**Table 1:** Summary of transcription factors up-regulated in module tan

Gene	Family	logFC	qPCR	Connectivity	Annotation
Potri.015G129100	MYB	1.520	2.497	0.982	myb domain protein 85
Potri.001G112200	HB-KNOX	1.556	3.416	0.980	KNOTTED-like homeobox of Arabidopsis thaliana 7
Potri.001G267300	MYB	2.023	1.726	0.974	myb domain protein 83
Potri.001G214700	YABBY	1.365	1.298	0.965	Plant-specific transcription factor YABBY family protein
Potri.015G082700	MYB	1.639	2.892	0.964	myb domain protein 50
Potri.002G031000	HB-BELL	1.773	1.402	0.962	BEL1-like homeodomain 7
Potri.005G118000	MADS	2.116	1.232	0.953	MADS-box transcription factor family protein
Potri.008G017500	OFP	2.889	3.028	0.939	Ovate family protein 13
Potri.007G032700	HB-BELL	1.351	2.816	0.929	BEL1-like homeodomain 4
Potri.017G016700	NAC	1.003	1.541	0.917	NAC domain-containing protein 73
Potri.010G099100	C2H2	1.645	2.641	0.912	Indeterminate(ID)-domain 5
Potri.014G057700	SBP	1.269	1.057	0.896	Squamosa promoter binding protein-like 8
Potri.012G084100	MYB	1.499	2.537	0.892	myb domain protein 86
Potri.003G132000	MYB	1.657	2.013	0.885	myb domain protein 103
Potri.002G008800	HB-WOX	1.049	1.348	0.880	WUSCHEL-related homeobox 13
Potri.005G129500	HB-BELL	1.278	2.663	0.856	BEL1-like homeodomain 4
Potri.001G276200	SRS	1.498	1.289	0.766	Lateral root primordium (LRP) protein-related
Potri.017G081700	HD-ZIP	1.956	3.092	0.745	Homeobox 3
Potri.017G044200	MADS	3.506	4.672	0.745	MADS-box transcription factor family protein
Potri.003G114100	MYB	2.208	4.852	0.725	myb domain protein 42
Potri.005G118200	SRS	2.616	3.784	0.717	Lateral root primordium (LRP) protein-related
Potri.001G120200	YABBY	2.824	2.076	0.714	Plant-specific transcription factor YABBY family protein
Potri.017G044500	MADS	1.990	3.617	0.712	MADS-box transcription factor family protein
Potri.013G156200	LBD	2.638	3.284	0.618	LOB domain-containing protein 15
Potri.001G118800	MYB	1.262	2.378	0.612	myb domain protein 42

### A BELL transcription factor participated in establishing the secondary vascular tissue

**Fig. 4:**
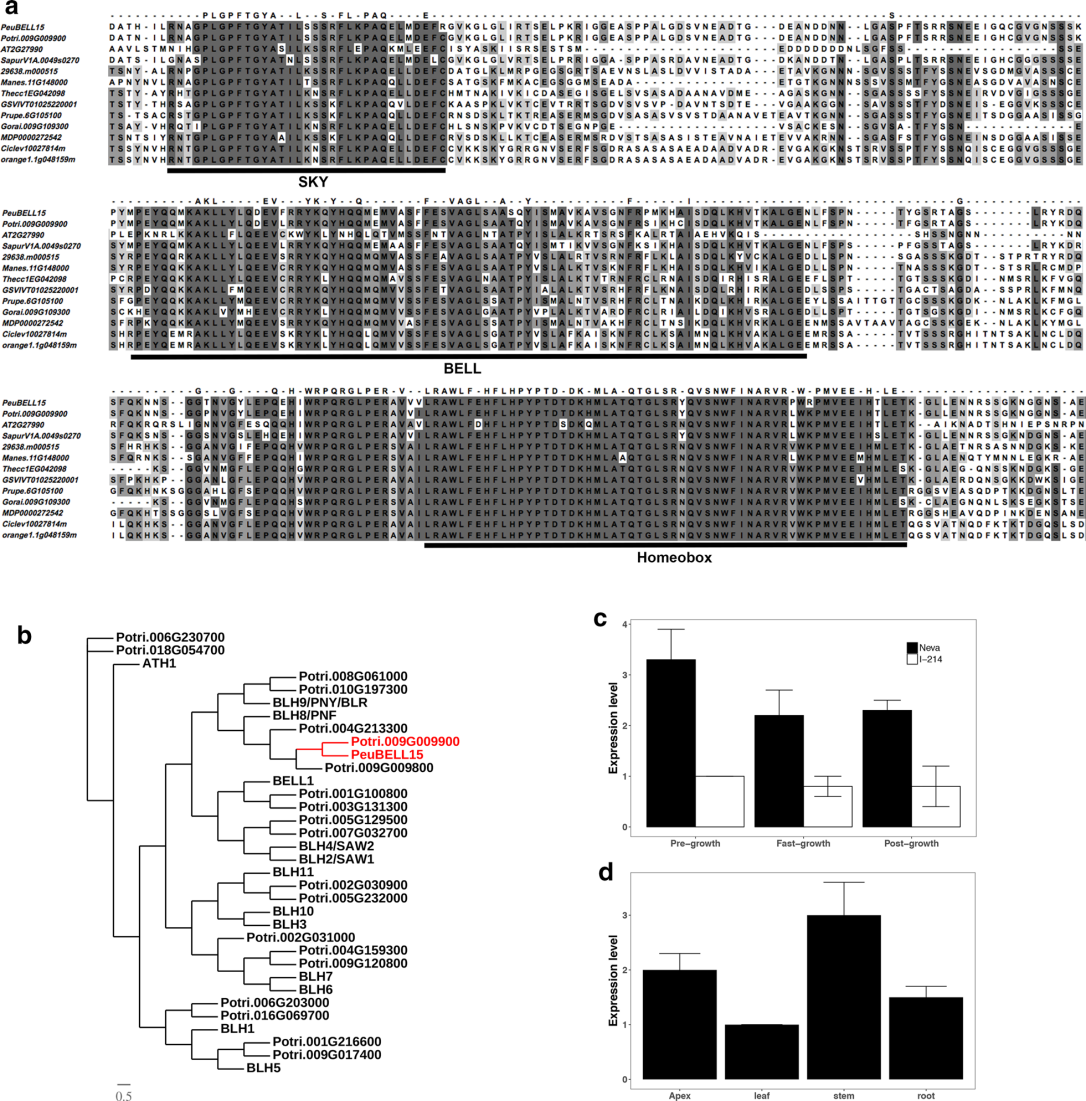
The expression pattern and phylogenetic relationships of *PeuBELL15*. **a** The protein sequence alignment of *BELL* genes from diverse species indicated the three conserved domains. These BELL genes are from *Populus euramericana* (*PeuBELL15*), *Populus trichocarpa* (Potri.009G009900), *Arabidopsis thaliana* (AT2G27999), *Salix purpurea* (SapurV1A.0049s0270), *Ricinus communis* (29638.m000515), *Manihot esculenta* (Manes.11G148000), *Theobroma cacao* (Thecc1EG042098), *Vitis vinifera* (GSVIVG01025220001), *Prunus persica* (Prupe.6G105100), *Gossypium raimondii* (Gorai.009G109300), *Malus domestica* (MDP0000272542), *Citrus clementina* (Ciclev10027814m), and *Citrus sinensis* (orange1.1g048159m). **b** The phylogenetic relationship among the BELL family genes in *Populus* and *Arabidopsis*. **c** The differential expression profiles of *Populus BELL15* in Neva and I-214 in different growth stages. **d** The tissue-specific expression pattern of *Populus BELL15*

To investigate the significance of genes identified by transcriptional profiles of *Populus* cambium in a more detailed context, we explored the potential functions of individual genes by overexpression and CRISPR/Cas9-induced knock-out mutation. We cloned a BEL1-like homeodomain (BELL) transcription factor gene *PeuBELL15*, the ortholog of *Populus trichocarpa PtrBELL15* (Potri.009G009900), from Neva (Genbank accession number is KY423502). The cDNA was 2436 bp long, and it encoded 811 amino acid residues with a predicted molecular weight of 1501.428 kDa and an isoelectric point of 6.3. The protein alignment presented three conserved motifs in BELL proteins from various species, including PeuBELL15 (Fig. 4a). The BELL transcription factors belong to the three amino acid loop extension (TALE) family [31]. The genome-wide survey investigated 35 *Populus* TALE genes presented, including 20 KNOX and 15 BELL family members (Additional file 13: Data S8). The phylogenetic analysis between *Populus* and *Arabidopsis* TALE factors showed a close relationship between *BLH8*/*PNF* (At2g27990) and *PeuBELL15* (Fig. 4b). The *PeuBELL15* gene showed higher expression levels throughout the growing season in Neva (Fig. 4c). Tissue-specific expression analysis showed that *PeuBELL15* gene mainly expressed in stems and the apex (Fig. 4d).

**Fig. 5:**
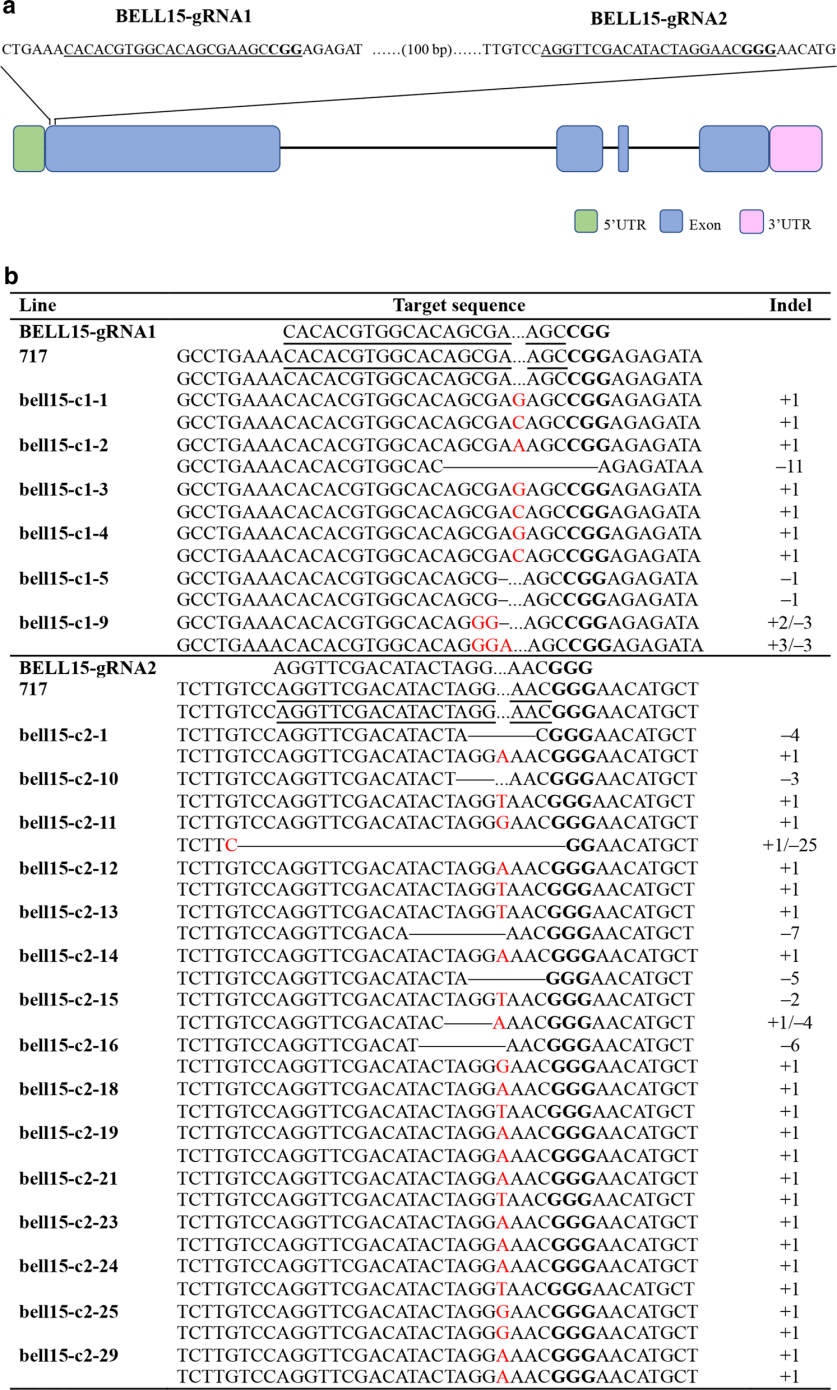
The targeted mutations of *Populus BELL15* mediated by CRISPR/Cas9 system. **a** Schematic illustration of target locations of CRISPR guide RNAs from *Populus BELL15*. **b** The mutagenesis of two target locations in *Populus BELL15* induced by CRISPR/Cas9 system

CRISPR has reformed the genome engineering world thanks to the ease and flexibility of using it. Although it has certain limitations, such as off-targets and chimeric mutations, it is still a powerful tool for modifying the genetic code and rendering a gene silent [32, 33]. It makes mutagenesis easier, especially for woody perennial plants with long generation periods. In this study, we used the CRISPR/Cas9 system to edit the genomic sequence of the ortholog of *PeuBELL15* in *Populus* clone 717. We designed two guide RNA (gRNA) targeting the first exon locus (Fig. 5a) to shift the reading frame. Sequencing results showed that these two gRNAs successfully modified the target loci by base deletion or insertion and they produced biallelic mutation (Fig. 5b). The mutation patterns of one-base insertion and deletion happened in most cases, and the indel patterns differed between alleles in many events.

**Fig. 6:**
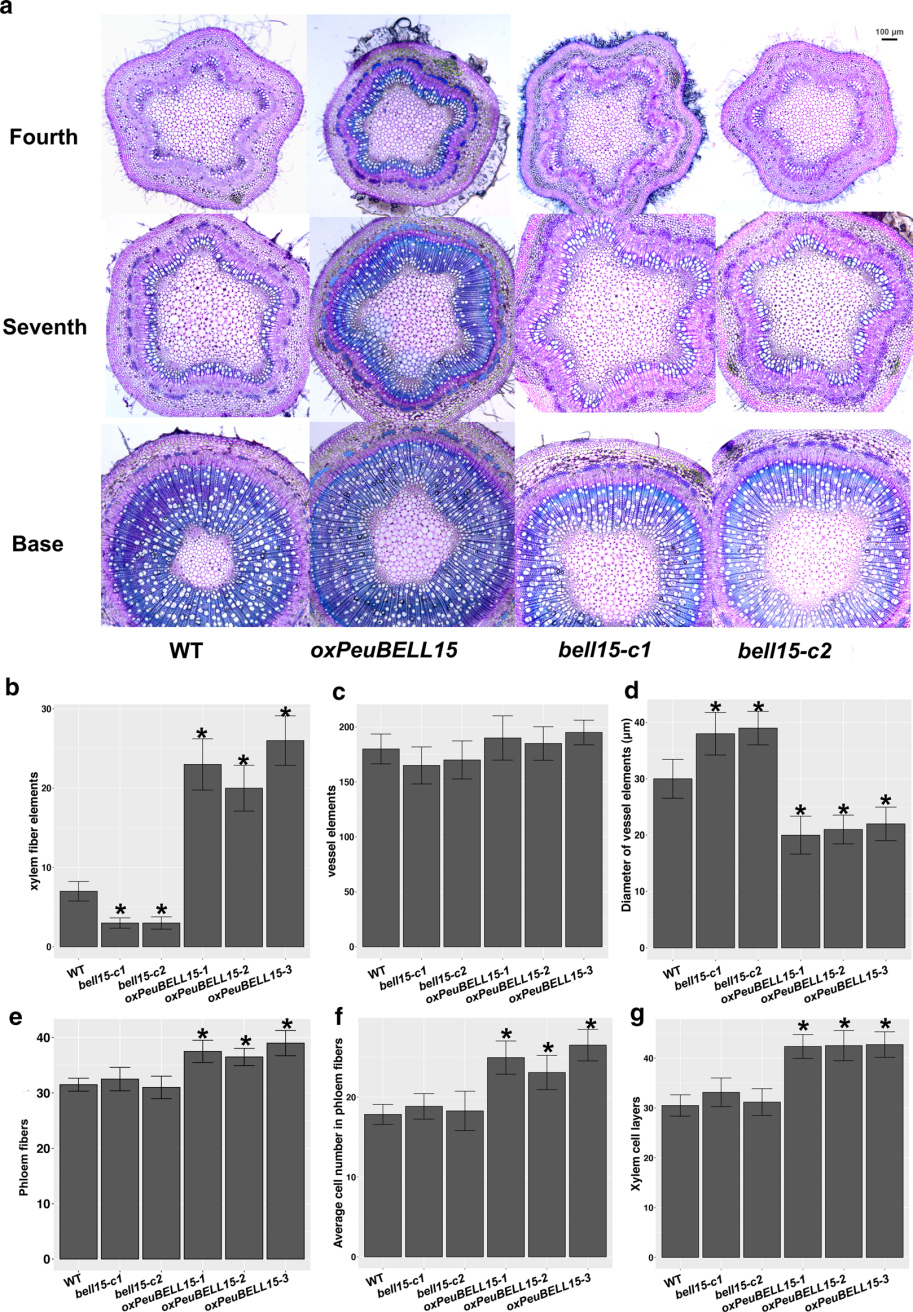
Changes in stem anatomy caused by mis-regulation of *PeuBELL15* in *Populus*. **a** Transverse sections of stems of wild type, *oxPeuBELL15*, and *bell15* mutant plants. **b** Xylem fiber elements of seventh internode. **c** Vessel elements of seventh internode. **d** Vessel size of seventh internode. **e** The phloem fibers and **f** average cell numbers in phloem fibers of wild type, *oxPeuBELL15*, and *bell15* mutant plants. **g** Xylem cell files of wild type, *oxPeuBELL15*, and *bell15* mutant plants

**Fig. 7:**
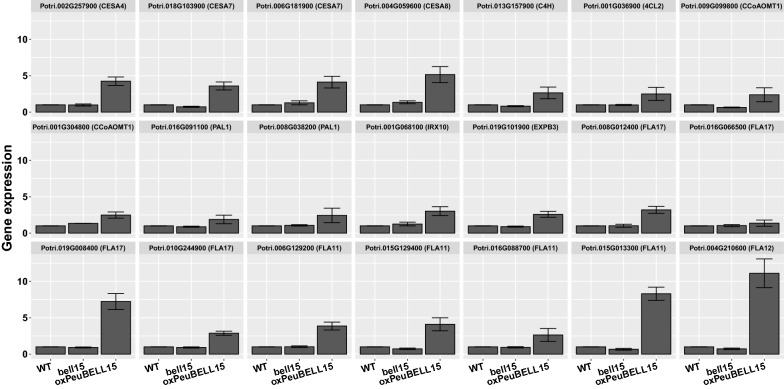
Expression profiles of genes related to secondary cell wall development mediated by *PeuBELL15* expression

**Fig. 8:**
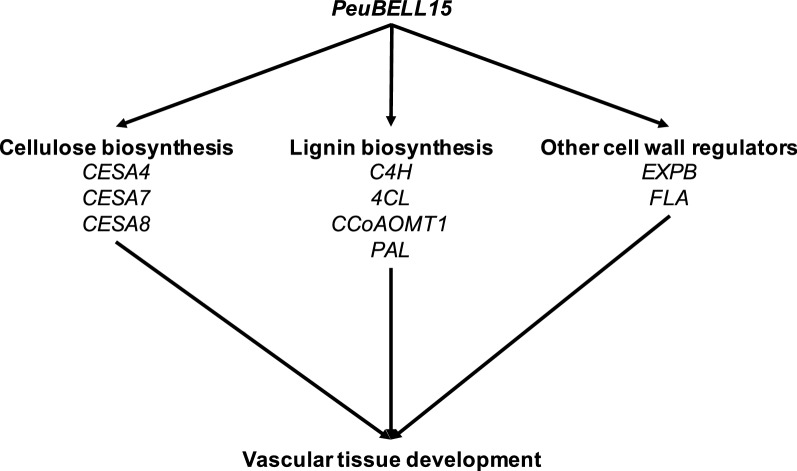
A model for the regulation of vascular tissue development by *PeuBELL15*. During the growth, *PeuBELL15* is expressed in the stem and up-regulates the expression levels of cellulose biosynthesis and lignin biosynthesis-relevant genes to supply compositions for secondary cell wall biosynthesis in vascular tissues. Additionally, expression abundances of other cell wall regulator genes such as *EXPB* (*β*-*expansin*) and *FLA* (*fasciclin*-*like arabinogalactan protein*) are increased by *PeuBELL15* to promote the cell expansion of vascular tissues

Although neither *PeuBELL15* overexpression (*oxPeuBELL15*) nor CRISPR/Cas9-mediated mutant (*bell15*-*c1*/*c2*) presented notable morphological variation with control plants (WT), transverse sections of stems revealed that *PeuBELL15* overexpression promoted the establishment of secondary vascular tissue elements (Fig. 6a). The transition from primary to secondary growth of vascular tissue was seen from the apex of the shoot to the bottom with the section stained with toluidine blue. At the fourth internode, the WT plants showed a transition to secondary growth and formed the cell files in the cambial zone. In the *oxPeuBELL15* plants, secondary phloem and xylem cells had been produced, and a continuous ring of secondary xylem cells appeared. In addition, precocious lignification was found in the secondary xylem cells and in phloem fibers. By the seventh internode, more extensive secondary xylem was present in *oxPeuBELL15* plants to increase the cell file layers, as were differential lignified phloem fibers. Whereas that matched WT plants showed only a continuous ring of secondary xylem. No highly lignified phloem fibers were in the same development stage. In addition, there were more xylem fiber elements in *oxPeuBELL15* than in WT. Vessel elements were not significantly increased but they were narrower in *oxPeuBELL15*. The mutant *bell15*-*c1/c2* plants presented fewer fiber elements and wider vessel elements compared with WT plants (Fig. 6b–d). At the bottom of the stem, the structure of the pith, successive layers of secondary xylem, cambial zone, secondary phloem with peripheral phloem fibers, cortex and an outer epidermis were all present in the plants. The *oxPeuBELL15* plants presented more secondary phloem fibers (Fig. 6e–f) and cell file layers of secondary xylem (Fig. 6g). Analysis of the wood chemical composition showed significant increases of glucan and lignin contents, but not for xylan in overexpressing plants (Table 2). Gene expression analysis showed that poplar orthologs of cellulose synthase *CESA4*, *CESA7*, and *CESA8* were up-regulated in *oxPeuBELL15* plants. Orthologs of genes participating in lignin biosynthesis, including *cinnamate*-*4*-*hydroxylase* (*C4H*), *4*-*coumarate:CoA ligase* (*4CL*), *caffeoyl*-*CoA O*-*methyltransferase 1* (*CCoAOMT1*) and *phenylalanine ammonia lyase* (*PAL*), also were up-regulated. Other induced genes related to secondary cell wall development were *IRX*, *β*-*expansin* (*EXPB*) and *fasciclin*-*like arabinogalactan protein* (*FLA*) (Fig. 7). In summary, *PeuBELL15* overexpression improved the biosynthesis of secondary cell wall components glucan and lignin, and it promoted the establishment of secondary vascular tissues by regulating the expression of relevant biosynthesis genes (Fig. 8).

**Table 2:** Analysis of the chemical composition of stems of WT, CRISPR, and overexpressing plants

Genotype	Glucan (%)	Xylan (%)	Lignin (%)
WT	38.14 ± 0.87	16.52 ± 0.09	25.78 ± 0.19
*bell15*-*c1*	36.81 ± 0.81	16.37 ± 0.49	25.91 ± 0.20
*Bell15*-*c2*	37.25 ± 1.01	16.17 ± 0.49	25.48 ± 0.22
*oxPeuBELL15*-*1*	41.34 ± 0.51*	17.12 ± 0.41	28.96 ± 0.17*
*oxPeuBELL15*-*2*	42.14 ± 0.55*	16.85 ± 0.23	27.09 ± 0.29*
*oxPeuBELL15*-*3*	43.72 ± 0.89*	17.07 ± 0.57	29.52 ± 0.20*

Asterisks denote significant differences according to one-way ANOVA test: *p *≤ 0.05

## Discussion

The rapid growth of *Populus* trees, including stem radial growth and shoot height growth, is central to effective production of woody biomass. Available data have shown that genetic differences could have a pronounced effect on diameter growth and biomass production [2]. In this study, we used an integrative approach to understand the role of gene expression in the dramatic differences in growth between *Populus × euramericana* hybrid clones Neva and I-214. We exploited the different phases of annual stem growth to assign genes representing different transcriptional clusters to various biological processes in these two genotypes. These clusters elucidated the variation of transcriptional programming during the growing season and the differences of gene expression profiles between fast- and slow-growing plants.

### Regulation of cell cycle and division are fundamental to differences in stem radial growth

The increased girth and size of plant stem tissues derive from secondary growth, which is driven principally by vascular cambium, and the stem growth rate is mainly an outcome of the rates of cell division of the cambium [5]. In our study, gene co-expression network analysis showed that numerous genes related to cell cycle and division presented expression patterns in accordance with the radial increment of stems and with the resource support of carbon and energy from photosynthesis (Figs. 1c, f, 4c). These genes participated in the fundamental growth and development processes in both Neva and I-214. Most of the hub genes which has a high module membership had roles in cell structure and membrane functions. The top hub gene with the highest module membership, *galactosyltransferase* (*GALT*; Potri.001G450200), catalyzed substances to produce arabinogalactan. This is an important component of polysaccharides in the cell wall, and it plays a vital role in cell division and growth [34]. Calmodulin is an important part of calcium signaling transduction [35], and the calmodulin-binding protein IQ-domain protein (IQD; Potri.003G042700) could participate in the calcium-dependent pathway and regulate the secondary cell wall biosynthesis [36, 37]. Receptor kinase proteins (RLK) are important components of the cell membrane, and three RLK genes *Leucine*-*Rich*-*Repeat* (*LRR*), *Somatic Embryogenesis Receptor*-*like Kinase* (*SERK1*) and *Inflorescence Meristem Receptor*-*like Kinase* (*IMK*) showed an identical expression pattern in accordance with variations in the growth rate, suggesting their functions in the cell membrane during the cell development and growth. DNA replication is the preliminary process of cell division. *Budding Uninhibited by benzimidazole*-*related* gene (*BUBR*; Potri.005G258300), *STICHEL* (Potri.003G044900), *MCM* (Potri.009G121500) and *RiboNucleotide Reductase* (*RNR*; Potri.005G087300) participate in a series of processes in DNA replication. In addition, hormone auxin could be transferred by efflux carrier protein PIN1 (Potri.016G035300) and activate downstream pathways by inducing the transcription regulator auxin-induced protein IAA13 (Potri.010G065200). The important cell cycle-related protein *Cyclin D3:2* (*CYCD3*;*2*; Potri.005G141900) also changed with variations in the growth rate in both Neva and I-214. The study in *Arabidopsis* reports that *CYCD3* genes regulate the cambial cell proliferation and secondary growth [38], suggesting conserved functions of *CYCD3* genes in cell division for *Populus* and *Arabidopsis* plants.

Although these two genotypes performed similar transcriptomic programming in a majority of cell activities, some biological pathways were regulated differently in different growth stages. MCM family genes form a complex to license the origin of DNA replication during the S phase [24]. *MCM6* overexpression can maintain normal growth of pea plants under stress and confer salinity stress tolerance without loss of yield [39]. Except for complex formation with other MCM genes, MCM6 even forms oligomer and it functions as DNA helices to regulate DNA replication [40]. In module blue, the majority of MCM genes forming the complex had high module membership but no significantly differential expression in the two genotypes. One exception was the *MCM6* gene (Potri.001G074000), which was significantly up-regulated in faster-growing Neva in the pre-growth phase. These results suggest that the MCM family genes functioned as a complex in both genotypes. Moreover, *MCM6* functioned independently to further improve the cell cycle activities in Neva.

When entering the fast-growth phase, cell activities became more lively, and the number of differentially expressed genes increased. Abundant membrane receptor protein kinases showed differential expression patterns. Leucine-rich-repeat receptor protein kinases (LRR-RLKs) represent the largest group of RLKs in plant genomes [41]. They are essential components of cell membranes, and they are involved in protein–protein interaction and various transmembrane signal transduction [42]. Ten out of 11 differentially expressed LRR-RLK genes were up-regulated in Neva, but their detailed functions were still unknown. By the post-growth stage, the growth rates of both genotypes gradually slowed and the number of DEGs also declined. The genes implicated in chromosome segregation, nuclear division and microtubule and cell wall movement were down-regulated, in accordance with the decreased growth rates.

### Advanced establishment of secondary vascular tissue promotes the woody growth

The cell wall is critical to plant growth and development, the maintenance of its morphological architecture and its biomass resource [4, 43]. Though plant morphology varies in thousands of forms, the elementary composition of plant bodies, the cell wall, is highly conserved. The primary cell wall could be expended with an extension of cell size. After the primary growth stops, the development of the secondary cell wall begins with deposition of polysaccharides, including cellulose, hemicellulose, and cell wall-encrusting substances like pectin and phenolic polymers such as lignin, contributing to more strength for plant support [44–47]. Gene co-expression network analysis shed light on the significantly differential expression patterns of cell wall-related genes clustered between two genotypes. Most of the DEGs were up-regulated in the pre-growth stage in Neva. The increased photosynthetic capability of Neva supported more carbohydrates and other metabolites for cell wall biosynthesis. The differential expression of genes associated with the series of cell wall biosynthesis promoted the construction of vascular tissues. The major component of the cell wall is cellulose, whose synthesis driven by cellulose synthases. The key synthases *CESA7* (Potri.006G181900 and Potri.018G103900) and *CESA8* (Potri.004G059600 and Potri.011G069600) in secondary cell wall biosynthesis showed up-regulation in Neva. Hemicellulose is a diverse group of polysaccharides, which are biosynthesized by a series of glycosyltransferases. The structural genes functioning in hemicellulose biosynthesis, *IRX7*/*FRA8*, *IRX8*, *IRX9* and *IRX10* were up-regulated in Neva in the pre-growth stage. The *IRX7*/*FRA8* [48] and *IRX10* [49, 50] encode a glucuronyltransferase and a glycosyltransferase required for glucuronoxylan biosynthesis. In addition, galacturonosyltransferases, GAUT12/IRX8, GAUT-like GALT1 and TBL3, have roles in pectin biosynthesis [51–53]. Lignin, cross-linked phenolic polymers in the vasculature, provides mechanical strength for plant growth. The important enzymes C4H and CCoAOMT1 in the phenylpropanoid pathway for lignin biosynthesis also were induced [54]. These key protein enzymes are essential for proper disposition of components in cell wall construction.

In addition, transcription factors are crucial regulators of the development and growth of vascular tissue. A large proportion of differentially expressed transcription factors was found in the pre-growth stage. The MYB transcription factors play crucial roles in vascular development and secondary cell wall biosynthesis [27, 55]. These cell wall-related transcription factors are involved in the activation of the secondary cell wall biosynthesis during vascular construction [56–58]. The top hub genes, *MYB85* and *MYB83*, had high-connectivity co-expression patterns with some *CESA* and *IRX* genes in cellulose synthesis. This suggests potential indirect or direct regulation between these transcription factors and synthase proteins.

### TALE transcription factors regulate vascular development and growth

TALE proteins are ubiquitous transcription factors in the homeobox superfamily. They include two subfamilies, KNOX and BELL, which can function as heterodimers [31]. A total of 35 *Populus* TALE transcription factors were expressed in our dataset, and they were clustered in different co-expression modules based on their expression patterns (Additional file 11: Data S6), suggesting the diverse roles of TALE factors in cambial activities and vascular development. Two *Populus* KNOX ARBORKNOX1 genes, *ARK1a* and *ARK1b* [59], orthologous to class I KNOX gene *SHOOT MERISTEMLESS* (*STM*), presented high gene connectivity in the module blue. This suggests that these two genes have high degrees of co-expression with other genes in the module blue. Ectopic expressions of *ARK1a* and *STM* in *Populus* present orthologous functions in delayed differentiation of cambial cells and development of secondary vascular tissues [60]. *ARK1a* and *ARK1b* showed increased expression levels from the pre-growth to the fast-growth stage, and they decreased by the post-growth stage, but there was no significant difference between the two genotypes. It has been suggested that functions of *ARK1a* and *ARK1b* in secondary vascular development are fundamental to both genotypes, but they do not contribute to the differences in the growth of vascular tissues. The *KNOTTED*-*like ARABIDOPSIS THALIANA 1* (*KNAT1*) also has been reported to have redundant functions in the differentiation of xylem fibers [61]. The *Populus* orthologous gene *ARK2* has also been found to inhibit differentiation of cambial daughter cell [62]. *ARK2* displayed high expression levels in both Neva and I-214, and it had gradually elevated levels through the growing season. This may be due to compensatory mechanisms for a drop of *ARK1* expression in the post-growth stage. Functional interpretations of TALE family genes in *Arabidopsis* and *Populus* have shown that class II KNOX gene *AtKNAT7* and *Populus* orthologous gene *PoptrKNAT* are transcription repressors for secondary cell wall biosynthesis [28]. *PoptrKNAT* presented a higher expression level in Neva in the pre-growth stage. This was in accordance with the expression pattern of another hub gene, *MYB85*, in module tan, which, by contrast, promotes lignin biosynthesis in vascular development [63]. These two transcription factors could regulate downstream genes related to secondary cell wall development and maintain homeostasis of the vascular meristem.

To date, there has been little report on the roles of the BELL family in regulating vascular tissue development. The interpretation of *Arabidopsis BELL* genes indicated only the functions of *BELL* gene *PENNYWISE* (*PNY*) and *PNF* in apical meristem development [21, 22] and control of floral patterning [64]. The *Populus BELL15*, the orthologous gene of *PNF*, presented significantly differential expression in two genotypes during the whole growing period. This stem tissue-specific expression pattern suggests a potential role for that gene in stem growth and vascular development. Ectopic expression of *PeuBELL15* shows increased lignin and cellulose biosynthesis and earlier secondary vascular tissue establishment. At the pre-growth stage, the increased expression abundance of *PeuBELL15* improved expression of secondary cell wall biosynthetic genes in faster-growing Neva. The modified transcriptional remodeling contributed to the biosynthesis and deposition of components of the secondary cell wall in Neva. This was consistent with up-regulation of genes concerning cellulose synthases and lignin biosynthesis and increased secondary fibers in phloem and xylem tissues of Neva. Indeed, *PeuBELL15* positively regulated expression of these relevant biosynthesis genes to increase the production of fiber elements. The decreased ratio of vessels to fibers induced by *PeuBELL15* overexpression promoted the accumulation of wood biomass in *Populus* plants.

## Conclusions

The fast growth of hybrid *Populus* × *euramericana* clones makes them important tree species for wood and biofuel production. The dynamic of cambial cells gives birth to the secondary vascular tissue, and it keeps the stem radial size of woody plants increasing year after year. We compared the cambial transcriptomic profiles of fast-growing genotype Neva and the control I-214 to discover the relationship between transcriptomic modification and variations in the growth rates of stem radial size. In the growing season, Neva presented more vigorous events of the cell cycle and division in the pre-growth and fast-growth phases, leading to more stem radial cumulation. Meanwhile, a BELL transcription factor PeuBELL15 was found to play positive roles in the improvement of vascular development. The early establishment of the mature vascular structure is more helpful to substance transportation and even signal transduction. Thus, it promotes the rapid growth and accumulation of biomass of plants. Generally speaking, the more active division and expansion of vascular cambium cells, secondary cell wall deposition of xylem cells, and earlier establishment of secondary vascular tissues lead to more stem radial increment and biomass accumulation. These interesting genes related to vascular development and growth will be targets for directional elite breeding of trees focusing on the accumulation of wood biomass.

## Methods

### Plant materials and growth conditions

The *Populus* plants used in the experiments were two hybrid clones of *Section Aigeiros*, Neva (*Populus* × *euramericana* cv ‘Neva’) and I-214 (*Populus*$\times$*euramericana* cv ‘I-214’). In early April 2013, stem cuttings (about 15 cm in height and 1.5 cm in diameter) of these two clones imbibed water for 48 h, then planted in pots that contained turfy, loamy soil and perlite (3:1:1, v/v/v) in the nursery of Beijing Forestry University (116.3 °E, 40.0 °N). The subject trees were irrigated every 3 days to provide vigorous growing conditions.

The genetic background of all trees used for CRISPR editing and transformation is hybrid aspen *Populus tremula *× *Populus alba* clone INRA 717-IB4. Plants used for histology were transferred to soil (Sunshine Mix 4; SunGro Horticulture) in 0.75-l pots, covered with a transparent lid (Super Sprouter), and adjusted to ambient humidity over 2 weeks. Plants were used for experiments after 2 months of growth in soil with water supplemented with Miracle Grow fertilizer using the concentration recommended by the manufacturer. Plants were cultivated at ~ 22 °C under continuous light provided by Philips TL830 and TL850 fluorescent bulbs.

### Physiological measurements

The stem radial growth of the two genotypes was measured in real-time and continuously during the growing season (from June to September). Well-growing and trunk-straight plants were selected to monitor the variation of stem growth by automated point dendrometer (Dendrometer DD-S, ECOMATIK, Munich, Germany), which was installed at the 15th node from the apical buds. A data logger (DL15, ECOMATIK, Munich, Germany) collected and recorded data every 15 min. Mature, fully expanded leaves of 20 plants from each genotype were selected randomly to determine the maximum photosynthetic rate and transpiration rate. The data were collected at an ambient CO_2_ concentration of 360 μmol mol^−1^ and photosynthetic photon flux density of 800 μmol m^−2^ s^−1^ using the LI-6400 portable photosynthesis system (Li-Cor Inc., Lincoln, NE, USA). Relative water content in the tree stems was recorded from the stem samples cut from the 15th node. The fresh weight (FW) was weighed immediately after collection and the dry weight (DW) was recorded after a stem was dried at 80 °C for 72 h. The stem water content (SWC) was calculated as SWC = (FW−DW)/FW [65].

### RNA extraction, illumina sequencing and data processing

The cambial region was harvested from the stem samples (~ 40 cm per tree) cut from the plant collar on three individual trees for each genotype on June 8, August 1 and September 18. Samples from each tree were frozen quickly in liquid nitrogen, to allow separation of the bark (cambium with differentiating xylem and phloem cells) from the mature xylem. After bark peeling, the white powdery cambial tissue was harvested by scraping the inner bark with a scalpel [12]. Powders of 18 samples were used for total RNA extraction by the Total RNA Purification Kit (TRK1001, LC Science, Houston, TX) following the manufacturer’s procedure. A Bioanalyzer 2100 and RNA 6000 Nano LabChip Kit (Agilent, CA, USA) were used to determine the quality and purity of total RNA samples with mean RNA integrity number (RIN) > 7.0. Approximately 10 μg of total RNA representing a specific sample was prepared according to the protocol for the RNA-Seq sample preparation kit (Illumina, San Diego, USA). The single-end sequencing was performed on an Illumina Hiseq 2000 following the vendor’s recommended protocol.

The raw data were initially cleaned by removing the adaptor sequences and reads with more than two unknown bases and low-quality bases (> 20% of the bases with a quality score Q ≤ 20). The program TopHat2 [66] with default parameters was used to align the clean reads from the 18 samples to the referenced *Populus trichocarpa* genome assembly V 3.0 (http://www.phytozome.net/poplar.php). No more than a 2-bp mismatch was allowed when considering differences between different *Populus* species. The reads mapped to multiple genes also were filtered out to obtain the unique clean reads used for further analyses. The unique mapped reads were used for count calculation by the HTSeq program [67]. The expression levels were calculated as reads per kilobase per million reads (RPKM), which were normalized by the TMM package [68]. The differentially expressed genes were identified using the edgeR package [69], and the statistical significance was evaluated by Fisher’s exact test with a false discovery rate (FDR) using the BH method to correct *p* value with a cut-off of 0.05. The genes with FDR < 0.05 and | log_2_ (fold change) | > 1 were considered as significantly DEGs. The DEGs with expression levels higher in Neva than in I-214 were described as up-regulated, and lower levels were termed as down-regulated. Functional annotation and enrichment of DEGs and co-expression modules through gene ontology enrichment were assigned using the GOstats and GSEABase packages [70] with *p* < 0.01. All statistical analyses were deployed in R unless stated otherwise.

### Gene co-expression network analysis

Co-expression and module analyses were executed using R package WGCNA V 1.51 [18]. Briefly, co-expression relationships between genes were summarized as an adjacency matrix and raised to the soft threshold power of 10. The soft threshold power was determined based on a greater 85% model fit to scale-free topology and low mean connectivity (Additional file 14: Figure S6). Co-expression modules were defined as clusters of highly interconnected genes and calculated using hierarchical clustering of dissimilarity of the measure of topological overlap. Hierarchical clustering was performed using the dynamic tree cutting with a minimum module size of 400 and a cut height of 0.994, and closely related modules (0.25) based on module eigengene values were collapsed. Traits were related to modules using module eigengene values. Module correlations for experimental treatments such as *Populus* genotypes and growth stage time points were tested by ANOVA. Hub genes in the module red were identified as genes that had high gene significance with genotype and high module membership in the module red. Gene significance was calculated as the correlation between gene expression RPKM and genotype. Module membership was calculated as the correlation between gene expression and module eigengene values for the module red. Analyses of the module blue and tan followed the same procedure.

### Identification of differentially expressed genes with real-time quantitative PCR (qPCR)

To confirm the expression patterns of the identified genes, a qPCR analysis was performed in representative genes, using the same RNA employed for transcriptome sequencing analysis. First-strand cDNA was synthesized starting from sample RNA, with AMV reverse transcriptase (Promega) following the manufacturer’s instructions. The primers were designed using Primer-BLAST software [71]. Primer names and nucleotide sequences are presented in Additional file 15: Data S9. The quantitative PCR reaction system (20 μl) included: template 1 μl (200 ng), forward primer 0.25 μl (20 μM), reverse primer 0.25 μl (20 μM), ddH_2_O 8.5 μl and SYBR qPCR Mix 10 μl. The reaction was amplified for 60 cycles each at 95 ℃ for 20 s, 60 ℃ for 35 s and 72 ℃ for 30 s. The PtUBQ gene was used for control.

### *PeuBELL15* gene cloning and transformation

The *PeuBELL15* gene was cloned from Neva. RNA extraction and subsequent cDNA synthesis from stems of *Populus* were performed as described above. The *PeuBELL15* coding sequence was amplified using specific primers (Additional file 15: Data S9). The coding sequence was introduced into the pK2GW7 expression vector by the Gateway strategy to construct a 35S promoter-driven overexpression cassette *35S:PeuBELL15*. The expression vector was transformed into *Agrobacterium tumefaciens* strain GV3101. The *Agrobacterium*-mediated transformation of *Populus* clone 717 was performed based on the referenced protocol [72]. The leaf discs were soaked for 1 h in resuspended *Agrobacterium* cells with *35S:PeuBELL15* vector. The inoculated leaf discs were co-cultivated at 22 ℃ in the dark for 2 days. Then they were transferred to the medium for callus inducement supplemented with 500 mg L^−1^ cefotaxime and 50 mg L^−1^ kanamycin for 10–30 days in the dark. Shoot and root regeneration was performed on the medium with 100 mg L^−1^ kanamycin for several weeks. Transgenic lines were identified by PCR and used for subsequent experimental analysis.

### CRISPR/Cas9-mediated targeted mutagenesis of *Populus BELL15*

Two single guide RNA (sgRNA) sequences for *Populus BELL15* in clone 717 were designed based on the SNP-bearing 717 genomic database [33]. The target sites of the designed sgRNA were confirmed by amplifying and sequencing (the primers are in Additional file 15: Data S9). The designed sgRNA was assembled into entry vector pEn-Chimera and made a construct with destination vector pDe-CAS9 [73], utilizing Gateway recombination cloning technology (Invitrogen, USA). The CRISPR/Cas9 construct was transformed into *Populus* clone 717 using the method mentioned above. After confirmation of positive transgenic plants, Sanger DNA sequencing of PCR amplicons was used to evaluate the editing conditions of CRISPR/Cas9 transfection.

### Histology

Stems were sectioned for histology using a Vibratome Series 1000 (Heath Company) to a thickness of 50 to 100 μm. Sections for anatomical assays were stained with toluidine blue (TBO) staining solution (0.9 M Tris, pH 6.8, 0.05% TBO) for 30 s before being viewed with a Leica DMB compound microscope using a Leica DFC450C digital camera with live image stitching using Leica Live Image Builder software.

### Analysis of chemical components

Stems of 2-month-old poplar seedlings were collected from 15 individuals in each line for composition analysis. The chemical compositions of samples (glucan, xylan, and lignin, % w/w) were analyzed by the standard procedure of the National Renewable Energy Laboratory [74]. Briefly, after two-step sulfuric acid hydrolysis (72% and 4% H_2_SO_4_, respectively), the sugars (glucose and xylose) in the hydrolyzate were quantitatively determined by HPLC (waters e2695, USA) equipped with a Shodex SH1011 column. 0.005 M H_2_SO_4_ was used as the mobile phase at a flow rate of 0.5 ml min^−1^ and the column temperature was 50 °C. The content of acid-insoluble lignin was determined using gravimetric analyses [75, 76].

## Supplementary information

**Additional file 1: Figure S1.** Comparison of vascular tissues and wood chemistry composition between Neva and I-214. (A) Transverse sections of different internodes in stems. (B) Wood chemistry composition of stems. Asterisks denote significant differences according to one-way ANOVA test: *p* ≤ 0.05.

**Additional file 2: Data S1.** Expressed genes identified in all growth stages of Neva and I-214.

**Additional file 3: Figure S2.** Differential expression profiles of genes between different growth phases and genotypes. In all plots, points in red represent significantly differential expressed genes with FDR < 0.05. Blue lines correspond to a threshold of twofold change in expression. (A) Genes differentially expressed in interphase I (the fast-growth vs the pre-growth stage) for Neva. (B) Genes differentially expressed in interphase II (the post-growth vs the fast-growth stage) for Neva. (C) Genes differentially expressed in interphase I (the fast-growth vs the pre-growth stage) for I-214. (D) Genes differentially expressed in interphase II (the post-growth vs the fast-growth stage) for I-214. (E) Genes differentially expressed in the pre-growth stage between Neva and I-214. (F) Genes differentially expressed in the fast-growth stage between Neva and I-214. (G) Genes differentially expressed in the post-growth stage between Neva and I-214.

**Additional file 4: Data S2.** Summary of differential expressed genes between two neighbouring growth stages for Neva and I-214.

**Additional file 5: Data S3.** Gene Ontology annotation and enrichment analysis of differential expressed genes between two neighbouring growth stages for Neva and I-214.

**Additional file 6: Figure S3.** Plots for Gene Ontology enrichment analysis of differential expressed genes between two neighbouring growth stages for Neva and I-214.

**Additional file 7: Data S4.** Summary of differential expressed genes between Neva and I-214 at different growth stages.

**Additional file 8: Data S5.** Gene Ontology annotation and enrichment analysis of differential expressed genes between Neva and I-214 at different growth stages.

**Additional file 9: Data S6.** Summary of differentially expressed transcription factors at different growth stages.

**Additional file 10: Data S7.** Gene Ontology annotation and enrichment analysis of genes clustered in each module.

**Additional file 11: Figure S4.** Enrichment of genes in module blue associated with gene ontology terms from cell cycle, cell division, chromosome, cytoskeleton, DNA, microtubule, nucleus, and organelle.

**Additional file 12: Figure S5.** The description of genes in module tan. (A) Correlation between module membership and differential expression of genes in module tan. Points in red refer to transcription factors. (B) The schematic map of the co-expression network of top hub genes in module tan.

**Additional file 13: Data S8.** Summary of *Populus* TALE transcription factors.

**Additional file 14: Figure S6.** Analysis of network topology for various soft-thresholding powers. (A) The scale-free fit index (y-axis) as a function of the soft-thresholding power (x-axis). (B) The mean connectivity (degree, y-axis) as a function of the soft-thresholding power (x-axis). In this study, we chose the power 10, which is the lower power for which the scale-free topology fit index curve flattened out upon reaching a high value of 0.85.

**Additional file 15: Data S9.** Primers used in this study for qPCR identification, gene cloning, and CRISPR vector construction.

## Data Availability

The raw data generated in this study have been deposited in the Short Read Archive in National Center for Biotechnology Information under the accession number SRP095978.

## Abbreviations

4CL: 4-Coumarate:CoA ligase
ARK: ARBORKNOX
BELL: BEL1-like
BUBR: Budding Uninhibited by Benzimidazole-Related
C4H: Cinnamate-4-hydroxylase
CAX: Calcium exchanger
CCoAOMT: Caffeoyl-CoA O-methyltransferase
CESA: Cellulose synthase
CNGC: Cyclic nucleotide-gated channel
CRISPR: Clustered regularly interspaced short palindromic repeats
CYCD: Cyclin D
DEG: Differentially expressed gene
DW: Dry weight
EXPB: β-expansin
FDR: False discovery rate
FLA: Fasciclin-like arabinogalactan protein
FW: Fresh weight
GALT: Galactosyltransferase
GAUT: Galacturonosyltransferase
GO: Gene ontology
IMK: Inflorescence meristem receptor-like kinase
IQD: IQ-domain
IRX: Irregular xylem
KNOX: KNOTTED-like
LRR: Leucine-rich repeat
LRR-RLK: Leucine-rich-repeat receptor protein kinase
MCM: Minichromosome maintenance
MDS: Maximum daily shrinkage
PAL: Phenylalanine ammonia lyase
PNF: POUND-FOOLISH
PNY: PENNYWISE
qPCR: Real-time quantitative PCR
RLK: Receptor kinase
RNR: Ribonucleotide reductase
RPA: Replication protein A
RPKM: Reads per kilobase per million reads
SERK: Somatic embryogenesis receptor-like kinase
sgRNA: Single guide RNA
STM: SHOOT MERISTEMLESS
SWC: Stem water content
TALE: Three amino acid loop extension
TBL: TRICHOME BIREFRINGENCE-like
WGCNA: Weighted gene co-expression network analysis
