# Correction to: Comparative transcriptome analyses define genes and gene modules differing between two *Populus* genotypes with contrasting stem growth rates

**DOI:** 10.1186/s13068-021-01910-4

**Published:** 2021-03-17

**Authors:** Xiao Han, Yi An, Yangyan Zhou, Chao Liu, Weilun Yin, Xinli Xia

**Affiliations:** 1 State Key Laboratory of Subtropical Silviculture, College of Forestry and Biotechnology, Zhejiang A&F University, Lin’an, Hangzhou, 311300 China; 2 Sino-Australia Plant Cell Wall Research Centre, State Key Laboratory of Subtropical Silviculture, College of Forestry and Biotechnology, Zhejiang A&F University, Lin’an, Hangzhou, 311300 China; 3 Beijing Advanced Innovation Center for Tree Breeding by Molecular Design, National Engineering Laboratory for Tree Breeding, College of Biological Sciences and Technology, Beijing Forestry University, Beijing, 100083 China

## Correction to: Biotechnol Biofuels (2020) 13:139 https://doi.org/10.1186/s13068-020-01758-0

Following publication of the original article [1], the authors would like to correct the funding statement. The correct number for the National Key Program on Transgenic Research is 2018ZX08020002. The number was changed from 2018ZX08021001 to 2018ZX08020002 during the project approval process. This has been corrected with this erratum.

The original article [1] has been updated.
