# Supplementary figures and images for: Comparative transcriptome analyses define genes and gene modules differing between two Populus genotypes with contrasting stem growth rates
Source: Biotechnol Biofuels. 2020 Aug 9;13:139. doi: 10.1186/s13068-020-01758-0 (PMC7415184; doi:10.1186/s13068-020-01758-0)

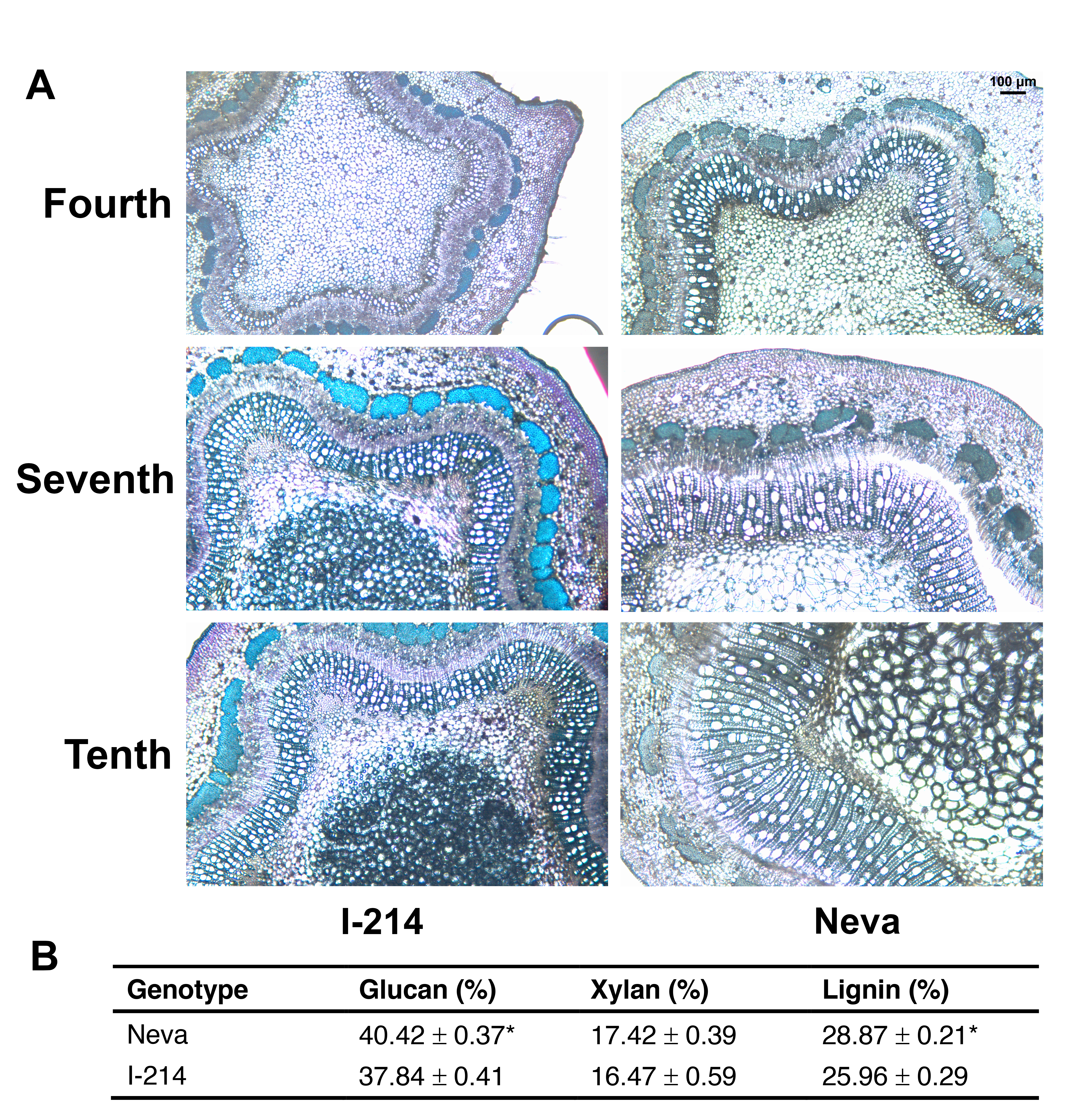

Supplement: Supplementary file 1 — Additional file 1: Figure S1. Comparison of vascular tissues and wood chemistry composition between Neva and I-214. (A) Transverse sections of different internodes in stems. (B) Wood chemistry composition of stems. Asterisks denote significant differences according to one-way ANOVA test: p ≤ 0.05. [file 13068_2020_1758_MOESM1_ESM.tif]

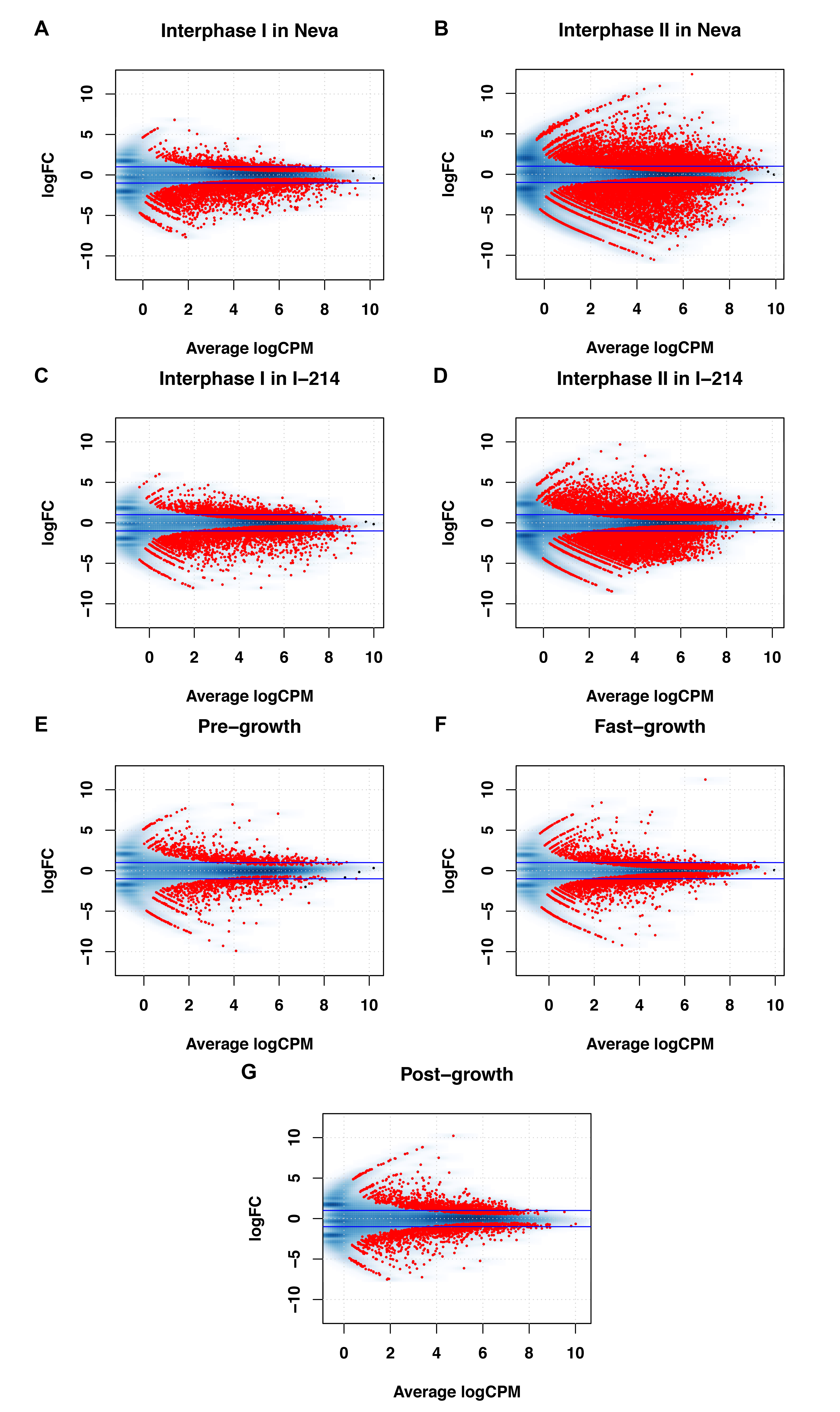

Supplement: Supplementary file 3 — Additional file 3: Figure S2. Differential expression profiles of genes between different growth phases and genotypes. In all plots, points in red represent significantly differential expressed genes with FDR < 0.05. Blue lines correspond to a threshold of twofold change in expression. (A) Genes differentially expressed in interphase I (the fast-growth vs the pre-growth stage) for Neva. (B) Genes differentially expressed in interphase II (the post-growth vs the fast-growth stage) for Neva. (C) Genes differentially expressed in interphase I (the fast-growth vs the pre-growth stage) for I-214. (D) Genes differentially expressed in interphase II (the post-growth vs the fast-growth stage) for I-214. (E) Genes differentially expressed in the pre-growth stage between Neva and I-214. (F) Genes differentially expressed in the fast-growth stage between Neva and I-214. (G) Genes differentially expressed in the post-growth stage between Neva and I-214. [file 13068_2020_1758_MOESM3_ESM.tif]

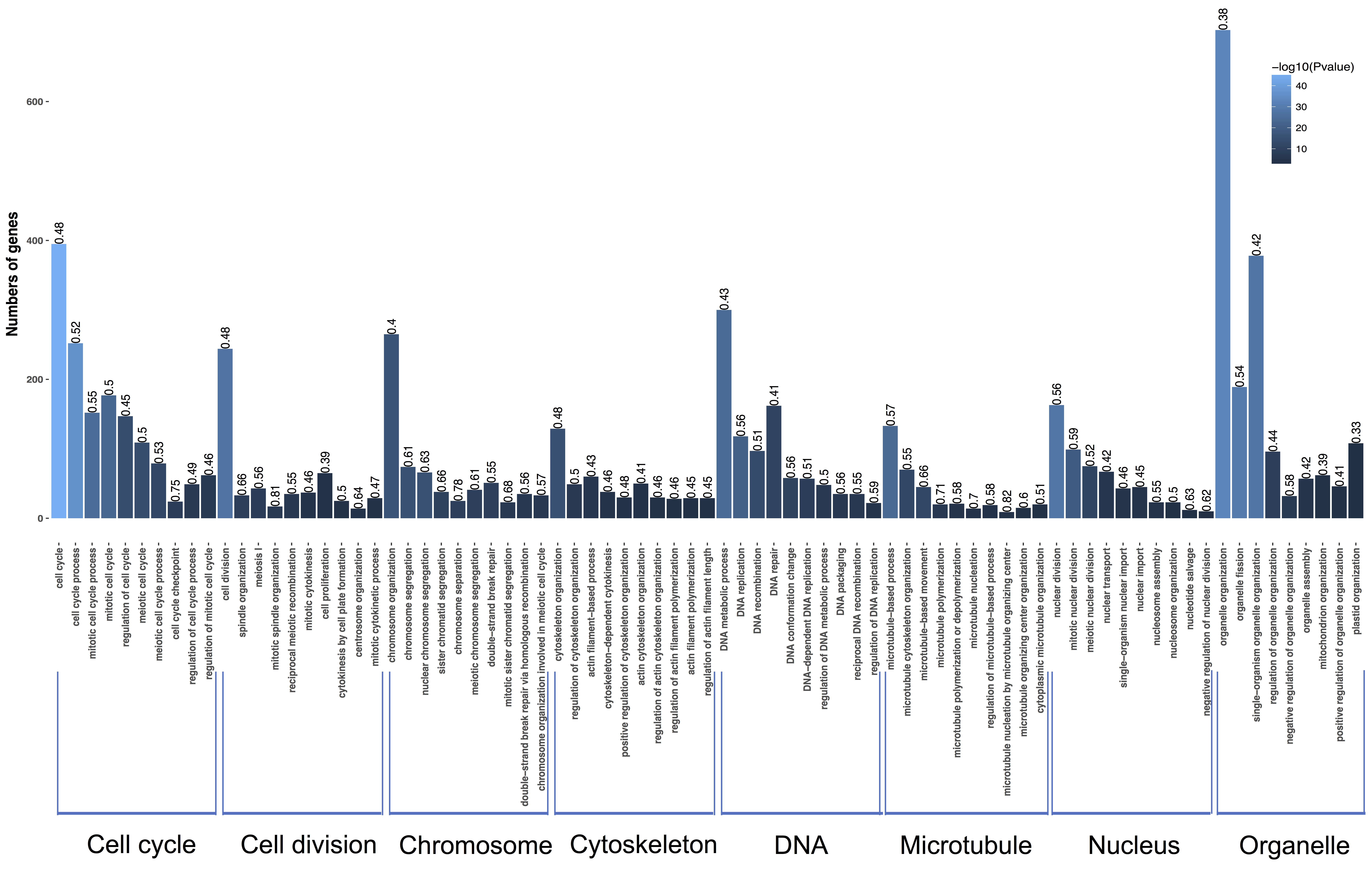

Supplement: Supplementary file 11 — Additional file 11: Figure S4. Enrichment of genes in module blue associated with gene ontology terms from cell cycle, cell division, chromosome, cytoskeleton, DNA, microtubule, nucleus, and organelle. [file 13068_2020_1758_MOESM11_ESM.tif]

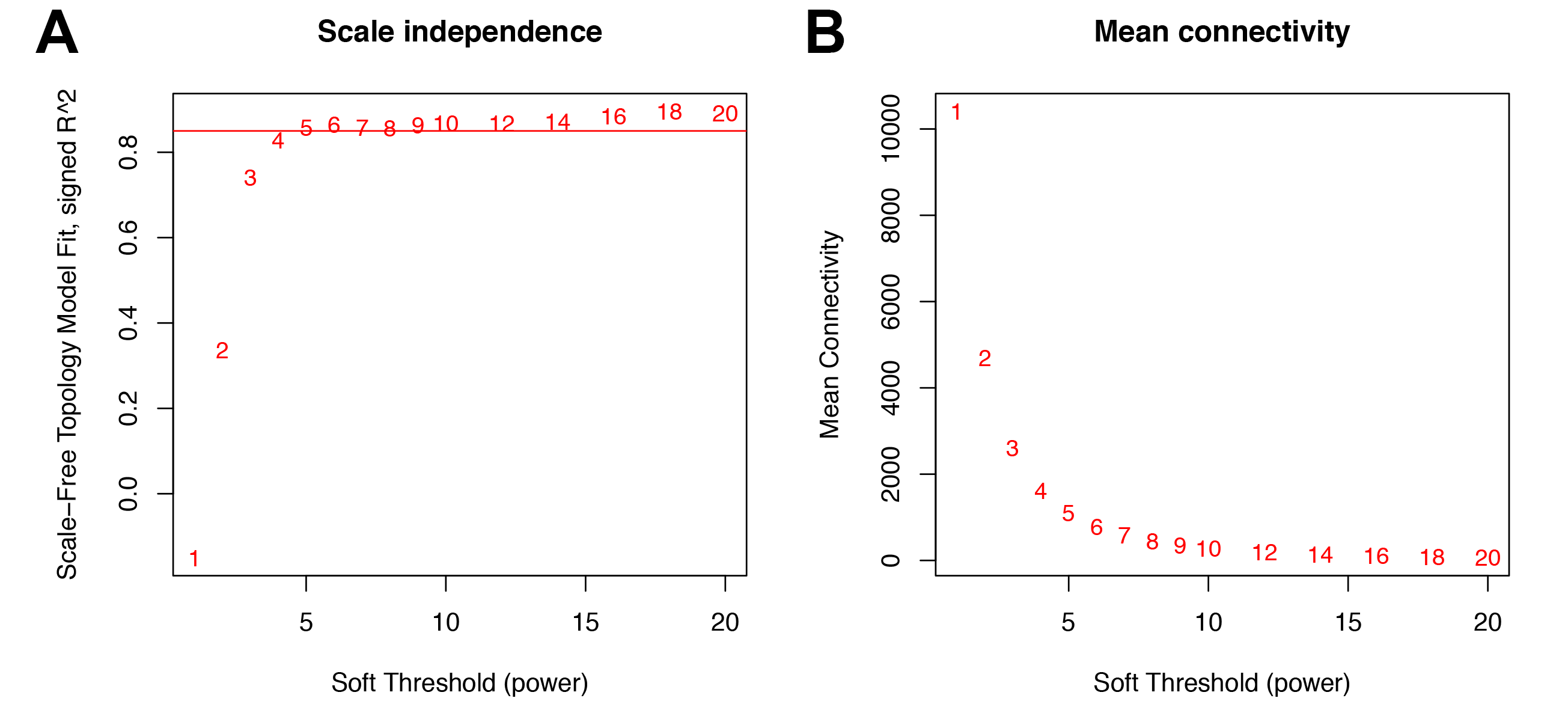

Supplement: Supplementary file 14 — Additional file 14: Figure S6. Analysis of network topology for various soft-thresholding powers. (A) The scale-free fit index (y-axis) as a function of the soft-thresholding power (x-axis). (B) The mean connectivity (degree, y-axis) as a function of the soft-thresholding power (x-axis). In this study, we chose the power 10, which is the lower power for which the scale-free topology fit index curve flattened out upon reaching a high value of 0.85. [file 13068_2020_1758_MOESM14_ESM.tif]
