# Supplementary material for: Comparative transcriptome analyses define genes and gene modules differing between two Populus genotypes with contrasting stem growth rates
Source: Biotechnol Biofuels. 2020 Aug 9;13:139. doi: 10.1186/s13068-020-01758-0 (PMC7415184; doi:10.1186/s13068-020-01758-0)
Supplement: Supplementary file 6 — Additional file 6: Figure S3. Plots for Gene Ontology enrichment analysis of differential expressed genes between two neighbouring growth stages for Neva and I-214. [file 13068_2020_1758_MOESM6_ESM.pdf]

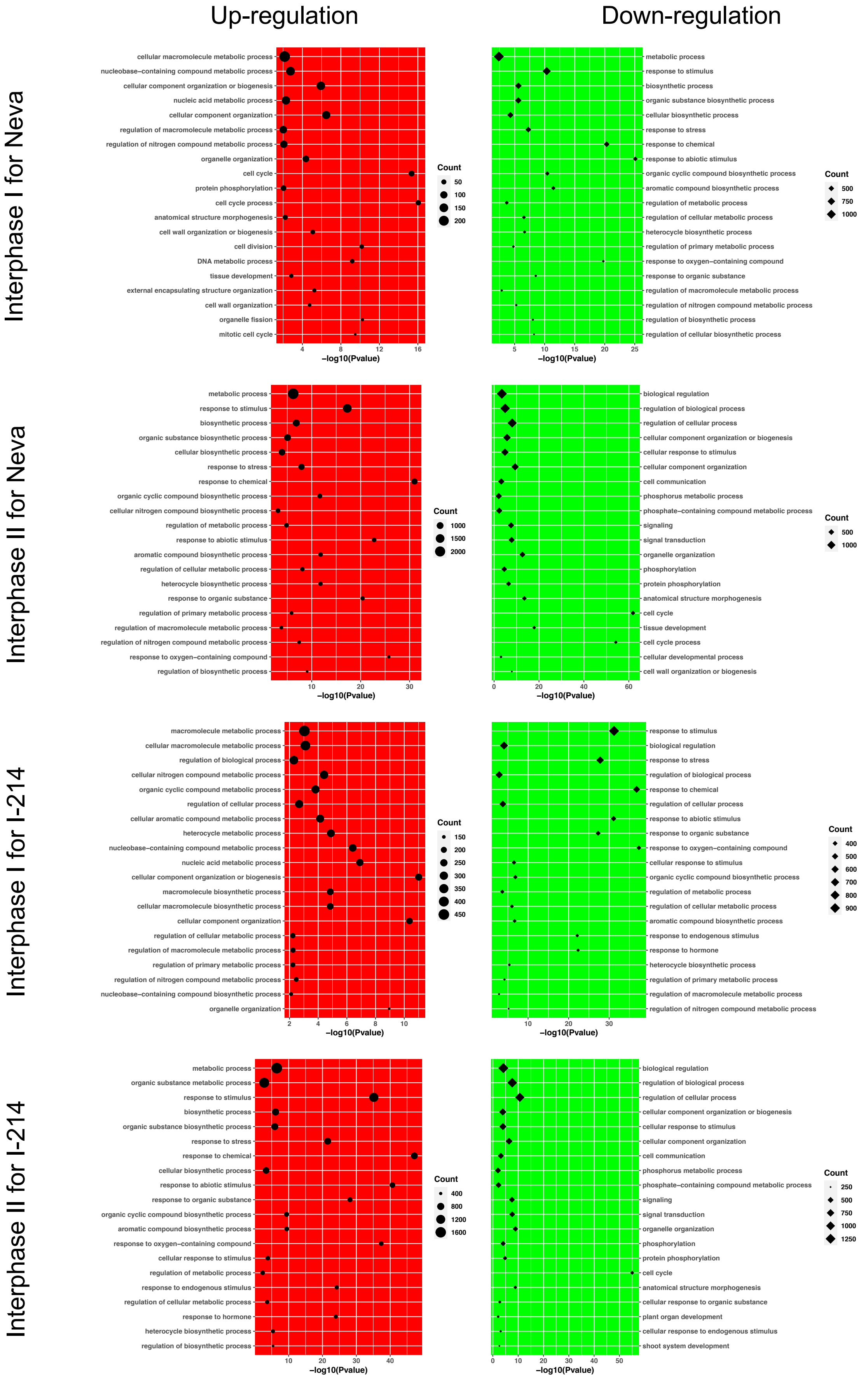
Up-regulation
Down-regulation
Interphase I for Neva
cellular macromolecule metabolic process
nucleobase−containing compound metabolic process
cellular component organization or biogenesis
nucleic acid metabolic process
cellular component organization
regulation of macromolecule metabolic process
regulation of nitrogen compound metabolic process
organelle organization
cell cycle
protein phosphorylation
cell cycle process
anatomical structure morphogenesis
cell wall organization or biogenesis
cell division
DNA metabolic process
tissue development
external encapsulating structure organization
cell wall organization
organelle fission
mitotic cell cycle
4 8 12 16
−log10(Pvalue)
Count
50
100
150
200
metabolic process
response to stimulus
biosynthetic process
organic substance biosynthetic process
cellular biosynthetic process
response to stress
response to chemical
response to abiotic stimulus
organic cyclic compound biosynthetic process
aromatic compound biosynthetic process
regulation of metabolic process
regulation of cellular metabolic process
heterocycle biosynthetic process
regulation of primary metabolic process
response to oxygen−containing compound
response to organic substance
regulation of macromolecule metabolic process
regulation of nitrogen compound metabolic process
regulation of biosynthetic process
regulation of cellular biosynthetic process
5 10 15 20 25
−log10(Pvalue)
Count
500
750
1000
Interphase II for Neva
metabolic process
response to stimulus
biosynthetic process
organic substance biosynthetic process
cellular biosynthetic process
response to stress
response to chemical
organic cyclic compound biosynthetic process
cellular nitrogen compound biosynthetic process
regulation of metabolic process
response to abiotic stimulus
aromatic compound biosynthetic process
regulation of cellular metabolic process
heterocycle biosynthetic process
response to organic substance
regulation of primary metabolic process
regulation of macromolecule metabolic process
regulation of nitrogen compound metabolic process
response to oxygen−containing compound
regulation of biosynthetic process
10 20 30
−log10(Pvalue)
Count
1000
1500
2000
biological regulation
regulation of biological process
regulation of cellular process
cellular component organization or biogenesis
cellular response to stimulus
cellular component organization
cell communication
phosphorus metabolic process
phosphate−containing compound metabolic process
signaling
signal transduction
organelle organization
phosphorylation
protein phosphorylation
anatomical structure morphogenesis
cell cycle
tissue development
cell cycle process
cellular developmental process
cell wall organization or biogenesis
0 20 40 60
−log10(Pvalue)
Count
500
1000
Interphase I for I-214
macromolecule metabolic process
cellular macromolecule metabolic process
regulation of biological process
cellular nitrogen compound metabolic process
organic cyclic compound metabolic process
regulation of cellular process
cellular aromatic compound metabolic process
heterocycle metabolic process
nucleobase−containing compound metabolic process
nucleic acid metabolic process
cellular component organization or biogenesis
macromolecule biosynthetic process
cellular macromolecule biosynthetic process
cellular component organization
regulation of cellular metabolic process
regulation of macromolecule metabolic process
regulation of primary metabolic process
regulation of nitrogen compound metabolic process
nucleobase−containing compound biosynthetic process
organelle organization
2 4 6 8 10
−log10(Pvalue)
Count
150
200
250
300
350
400
450
response to stimulus
biological regulation
response to stress
regulation of biological process
response to chemical
regulation of cellular process
response to abiotic stimulus
response to organic substance
response to oxygen−containing compound
cellular response to stimulus
organic cyclic compound biosynthetic process
regulation of metabolic process
regulation of cellular metabolic process
aromatic compound biosynthetic process
response to endogenous stimulus
response to hormone
heterocycle biosynthetic process
regulation of primary metabolic process
regulation of macromolecule metabolic process
regulation of nitrogen compound metabolic process
10 20 30
−log10(Pvalue)
Count
400
500
600
700
800
900
Interphase II for I-214
metabolic process
organic substance metabolic process
response to stimulus
biosynthetic process
organic substance biosynthetic process
response to stress
response to chemical
cellular biosynthetic process
response to abiotic stimulus
response to organic substance
organic cyclic compound biosynthetic process
aromatic compound biosynthetic process
response to oxygen−containing compound
cellular response to stimulus
regulation of metabolic process
response to endogenous stimulus
regulation of cellular metabolic process
response to hormone
heterocycle biosynthetic process
regulation of biosynthetic process
10 20 30 40
−log10(Pvalue)
Count
400
800
1200
1600
biological regulation
regulation of biological process
regulation of cellular process
cellular component organization or biogenesis
cellular response to stimulus
cellular component organization
cell communication
phosphorus metabolic process
phosphate−containing compound metabolic process
signaling
signal transduction
organelle organization
phosphorylation
protein phosphorylation
cell cycle
anatomical structure morphogenesis
cellular response to organic substance
plant organ development
cellular response to endogenous stimulus
shoot system development
0 10 20 30 40 50
−log10(Pvalue)
Count
250
500
750
1000
1250
